# Targeting the muscle-brain axis to improve post-stroke cognition via the FNDC5/irisin/BDNF pathway

**DOI:** 10.1016/j.jare.2025.10.027

**Published:** 2025-10-25

**Authors:** Jiating Wei, Yuangui Cai, Zimu Jiang, Jia Xie, Dingxiang Xie, Fubing Ouyang, Jianle Li, Zhiyi Xiong, Xiya Long, Miaoxian Yang, Lisi Zha, Yingxin He, Weixian Huang, Jinsheng Zeng

**Affiliations:** a Department of Neurology, The First Affiliated Hospital of Sun Yat-sen University, Guangzhou, Guangdong 510080, PR China; b Guangdong Provincial Key Laboratory of Diagnosis and Treatment of Major Neurological Diseases, Guangzhou, Guangdong 510080, PR China; c National Key Clinical Department and Key Discipline of Neurology, Guangzhou, Guangdong 510080, PR China; d Department of Radiology, The First Affiliated Hospital of Sun Yat-Sen University, Guangzhou, Guangdong 510080, PR China

**Keywords:** Post-stroke cognitive impairment, Muscle atrophy, Irisin, Exercise

## Abstract

**Introduction:**

The muscle–brain axis involving FNDC5/irisin/BDNF supports brain health and cognition. Stroke often causes hemiplegic muscle atrophy, which may disrupt this axis and impair cognition.

**Objectives:**

To determine whether stroke-induced muscle atrophy disrupts the FNDC5/irisin/BDNF axis and contributes to post-stroke cognitive impairment (PSCI) in patients, cynomolgus monkeys and rats.

**Methods:**

We conducted a cross-sectional study of stroke patients 3–6 months post-onset from outpatient (*n* = 24) and matched healthy controls from the community (*n* = 22). Cognitive function was assessed using the MoCA, TMT-A, TMT-B, and AVLT-H. Serum irisin was quantified using ELISA. Bilateral mid-thigh MRI scanning (mDIXON-QUANT) evaluated muscle cross-sectional area and fat fraction. Next, in stroke cynomolgus monkeys and rats, muscle atrophy, FNDC5/irisin/BDNF expression, and cognition were measured. Finally, we evaluated whether upregulation of the FNDC5/irisin/BDNF axis via physical exercise or peripheral irisin overexpression alleviated PSCI in rats.

**Results:**

Stroke patients exhibited a 6.5 % decrease in cross-sectional area (CSA) and a 13.6 % increase in fat fraction in the paretic thigh compared with the non-paretic side. Serum irisin levels dropped by 40 % compared to controls and were positively linked to paretic muscle CSA and cognitive function. In cynomolgus monkeys, paretic muscle CSA fell by 40 % at 12 weeks post-stroke, along with a notable decrease in PGC-1α and FNDC5 protein in atrophic muscle. These monkeys also exhibited a 50 % decrease in serum irisin levels, reduced hippocampal BDNF expression, and fewer immature neurons compared to sham-operated controls. Similarly, stroke rats demonstrated paretic muscle atrophy and suppression of the FNDC5/irisin/BDNF signaling pathway. Enhancing irisin via exercise or peripheral irisin overexpression significantly increased BDNF expression and immature neurons in the hippocampus of stroke rats, ultimately improving PSCI (all *p* < 0.05).

**Conclusions:**

Hemiplegic muscle atrophy contributes to PSCI through the FNDC5/irisin/BDNF axis downregulation. Elevating irisin shows therapeutic potential for PSCI.

## Highlights

• Serum irisin levels are reduced in stroke patients.
• Serum irisin levels correlates with muscle size and post-stroke cognition in patients.
• Muscle atrophy is accompanied with downregulation of FNDC5/irisin/BDNF axis in stroke monkeys and rats.
• Physical exercise or peripheral FNDC5/irisin overexpression restores the muscle-brain axis.
• Restoration of the muscle-brain axis improves post-stroke cognition.

## Graphical abstract

**Figure:**
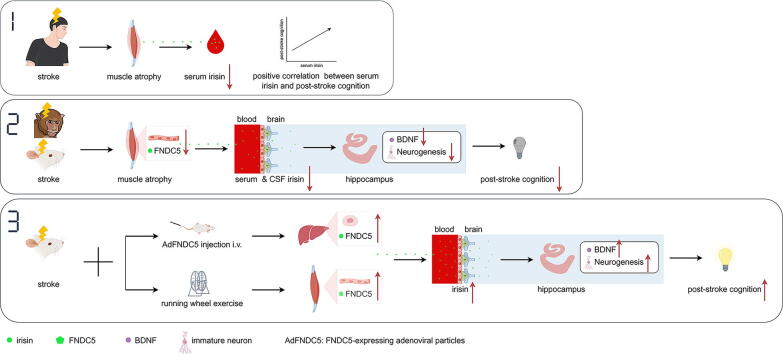

## Introduction

Post-stroke cognitive impairment (PSCI) poses a significant burden on stroke survivors. The decline in cognitive function may persist for as long as 12 years following the onset of the stroke, with approximately 20 % of stroke survivors eventually developing post-stroke dementia [1,2]. This prolonged decline offers a potentially valuable opportunity for therapeutic intervention. Several mechanisms, including cerebral small vessel disease and stroke-related secondary degeneration, have been implicated in the cognitive deterioration after stroke [3,4]. However, the precise mechanisms underlying cognitive decline in the chronic phase remain incompletely understood, and effective treatments for PSCI are currently lacking.

Traditionally, the skeletal muscle has been considered solely as an effector organ susceptible to stroke-induced damage. Chronic stroke patients exhibit a 24 % reduction in paretic thigh muscle volume compared to the contralateral side [5]. It is now widely recognized that skeletal muscle functions not only as a locomotor organ but also as an endocrine organ, releasing signaling molecules called myokines. These myokines enable skeletal muscles to have beneficial effects on brain health and cognition [6]. Sarcopenia, which involves the age-related decline in skeletal muscle mass, strength, and physical performance, is associated with cognitive impairment. It has been identified as an independent factor contributing to cognitive decline in older adults [7,8]. However, the potential impact of stroke-induced muscle atrophy on PSCI has not been extensively studied.

Irisin has been identified as a novel myokine that facilitates communication between skeletal muscles and the brain, promoting cognition [6]. It is generated through proteolytic cleavage from the N-terminal segment of fibronectin domain III containing 5 (FNDC5), a membrane-bound protein in skeletal muscle, and subsequently released into circulation. Studies have shown a positive correlation between serum irisin levels and global cognition as well as episodic memory in older individuals [9]. Additionally, compared with healthy controls, irisin levels in the cerebrospinal fluid (CSF) are significantly reduced in both Alzheimer’s disease patients and mouse models of Alzheimer’s disease [10]. Further studies have confirmed that peripheral delivery of irisin rescues the loss of long-term potentiation and memory in mouse models of Alzheimer’s disease by inducing brain-derived neurotrophic factor (BDNF) in the hippocampus [[10], [11], [12]]. The expression level of FNDC5/irisin mainly depends on muscle mass and exercise [[13], [14], [15]]. However, it remains unknown how stroke affects irisin production and whether alterations in this pathway contribute to PSCI.

Therefore, we hypothesized that stroke-induced hemiplegic muscle atrophy reduces FNDC5/irisin expression, leading to the downregulation of the FNDC5/irisin/BDNF axis and subsequent cognitive decline. To test this hypothesis, we initially quantified skeletal muscle atrophy and serum irisin levels in stroke patients, and examined the relationship between serum irisin levels and PSCI. Leveraging the close genetic and anatomical parallels between primates and humans, we then characterized changes in muscle morphology and FNDC5/irisin/BDNF signaling after chronic stroke in cynomolgus monkeys. Subsequently, we observed whether the FNDC5/irisin/BDNF axis exhibited similar changes after stroke in rats and cynomolgus monkeys. Finally, we assessed whether enhancing serum irisin levels through physical exercise or peripheral irisin overexpression could restore the FNDC5/irisin/BDNF axis and mitigate PSCI in stroke rats.

## Materials and methods

### Patient study

This study was approved by the Independent Ethics Committee of the First Affiliated Hospital of Sun Yat-sen University (FAH-SYSU-IEC-[2023]838). All participants provided written informed consent prior to participation. The study population consisted of patients who were hospitalized for acute ischemic stroke in the Department of Neurology, The First Affiliated Hospital of Sun Yat-sen University, and subsequently followed up at the neurology outpatient clinic 3 to 6 months after stroke onset. Inclusion criteria were: (1) aged 45 years or older; (2) first-ever ischemic stroke in the middle cerebral artery territory confirmed by imaging; (3) three to six months post-stroke, with residual motor deficits (Medical Research Council grade 3–4, modified Rankin Scale 1–4). Exclusion criteria were: (1) presence of other conditions affecting motor function, such as intracerebral hemorrhage and Parkinson's disease; (2) pre-stroke diagnosis of cognitive impairment or dementia; (3) other conditions leading to muscle atrophy; (4) presence of a brain tumor; (5) severe aphasia or mental disorder preventing completion of cognitive assessment; (6) heart failure, liver failure, renal failure or malignancy; (7) lack of informed consent; (8) contraindications for MRI. Healthy controls matched for age and vascular risk factors were recruited from the community through advertisements. The sample size was determined primarily by patient availability within the study period rather than a priori power calculation.

Bilateral thigh MRI was conducted using a 3.0 T MR scanner (Philips Ingenia, Philips Medical Systems, Netherlands). The mDIXON-QUANT sequence was utilized to evaluate the cross-sectional area (CSA) and fat fraction (FF) of the skeletal muscle in the bilateral mid-thigh. This sequence generated water phase, fat phase, in-phase, out-phase, and fat fraction images [16]. Regions of interest (ROI), including the vastus lateralis, vastus medialis, vastus intermedius, rectus femoris, sartorius, gracilis, adductor magnus, adductor longus, biceps femoris, semitendinosus, and semimembranosus, were manually segmented on the out-phase image at the mid-thigh level by an experienced radiologist blinded to patient information. Areas of suspected muscle strain, vascular, or fatty regions were excluded from the analysis. The ROIs from the out-of-phase images were transferred to the fat fraction images to quantify the CSA and fat fraction. Participants were all instructed to refrain from physical exercise on the day prior to fasting blood collection. Blood samples were obtained during the MRI visit and centrifuged at 3000 × rpm at 4 °C for 10 min to isolate the serum, which was stored at −80 °C until further analysis. Serum irisin levels were measured using a Human Irisin ELISA kit (CSB-EQ027943HU; CUSABIO). Clinical cognitive assessments were performed during the thigh MRI visit, including the Montreal Cognitive Assessment (MoCA), Trail Making Test Part A (TMT-A) and Part B (TMT-B), and Auditory Verbal Learning Test-HuaShan version (AVLT-H) for evaluating global cognition, executive function, and memory [17]. Clinical characteristics, such as age, sex, education, history of hypertension, diabetes, dyslipidemia, smoking, alcohol consumption, dysphasia, and serum albumin levels, were obtained from hospital records.

### Animals and experimental groups

All animal experimental procedures were conducted in accordance with the ARRIVE guidelines for the care and use of laboratory animals. Cynomolgus monkey study was reviewed and approved by the Institutional Animal Care and Use Committee of Guangdong Landau Biotechnology Co. Ltd. (LDACU20190215-01). SD rat study received approval from the Institutional Animal Care and Use Committee of Sun Yat-sen University (SYSU-IACUC-2023–001422). Six male cynomolgus monkeys (aged 4–5 years, weighing 5.5–6.0 kg) were provided by Guangdong Landau Biotechnology Co., Ltd. (Guangzhou, China), and 90 wild-type male Sprague Dawley (SD) rats (250–300 g, 10–12 weeks old) were purchased from the Animal Center of Sun Yat-sen University. All animals were housed under controlled conditions, including a temperature of 22 °C ± 2 °C, relative humidity of 60 % ± 5 %, and a 12-hour light/dark cycle. Cynomolgus monkeys were fed commercial monkey chews (Guangzhou Feed Research Institute, Guangzhou, China) supplemented with various vegetables and fruits twice a day. SD rats were provided with free access to standard laboratory chow and tap water. Cynomolgus monkeys randomly underwent middle cerebral artery occlusion (MCAO) or a sham operation (n = 3 per group). SD rats were randomly assigned to nine groups as follows: 1) sham, 2) sham + vehicle, 3) sham + anti-FNDC5, 4) MCAO, 5) MCAO + Exercise, 6) MCAO + Exercise + vehicle, 7) MCAO + Exercise + anti-FNDC5, 8) MCAO + AdGFP, and 9) MCAO + AdFNDC5 (*n* = 10 per group). Outcome assessment and data analysis were conducted by experimenters who were blinded to group allocation.

### MCAO in cynomolgus monkeys and rats

MCAO was conducted in cynomolgus monkeys following the methodology outlined in our previous study [18]. Briefly, anesthesia was induced through an intramuscular injection of ketamine (10 mg/kg) and maintained with isoflurane inhalation (1.5 % to 3 %) mixed with oxygen. Subsequently, a pterional craniotomy was performed to carefully expose and occlude the distal M1 segment of the middle cerebral artery using bipolar electrocoagulation. The occluded MCA was excised to prevent recanalization. Finally, the bone window was sealed with wax, and the incision was sutured. In the sham group, the MCA was exposed without occlusion. All monkeys survived the stroke until the time of sacrifice.

To induce MCAO in SD rats, the rats were anesthetized with 4 % isoflurane, followed by maintenance with 2 % isoflurane. Subsequently, the right common carotid artery (CCA), external carotid artery (ECA), and internal carotid artery (ICA) were exposed, and a nick was made in the distal of the ECA. A nylon thread was then inserted approximately 20 mm from the ECA into the ICA until encountering resistance. After two hours, the nylon thread was withdrawn for reperfusion [19]. The sham rats underwent an identical surgical procedure, excluding the filament insertion. Rats that experienced severe weight loss were euthanized and excluded if they did not survive within the first seven days post-stroke.

To avoid potential confounders, monkey and rat MCAO were performed by the same skilled operator.

### Physical exercise training protocol in rats

Rats in the MCAO + Exercise group began the running wheel exercise (RWE) seven days post-stroke using a programmable motorized wheel apparatus (21 cm in diameter, 40 cm in length) built at the South China University of Technology, Guangzhou, China. The running speed was incrementally raised to 10 rev/min during the initial week for acclimatization, as detailed previously [20]. The rats underwent training sessions twice daily, each lasting 30 min, for a duration of three weeks.

### Intravenous injection of neutralizing anti-FNDC5 antibody

Beginning on postoperative day 7, rats in the sham + vehicle, sham + anti-FNDC5, MCAO + Exercise + vehicle, and MCAO + Exercise + anti-FNDC5 groups received intravenous tail-vein injections of vehicle or anti-FNDC5 twice weekly for 3 weeks.

### Peripheral overexpression of FNDC5/irisin by adenoviral vectors in rats

High-titer GFP- or FNDC5-expressing adenoviral particles were generated by GeneChem (Shanghai, China). Seven days post-MCAO, animals in the MCAO + AdGFP/FNDC5 group received intravenous injections of GFP- or FNDC5-expressing adenoviral particles (5 × 10^9^/animal) via the caudal vein every 7 days for 3 weeks following the stroke.

### MRI of cynomolgus monkeys

MRI scanning was performed before and 12 weeks after MCAO in the monkeys, using a Siemens 3.0-T Prisma system with a homemade head coil. Time-of-flight magnetic resonance angiography (TOF-MRA) was performed to confirm occlusion. T2 fluid-attenuated inversion recovery (T2 FLIRA) was conducted to visualize the focal infarct.

### Neurological assessment in cynomolgus monkeys and rats

Neurological deficits in the cynomolgus monkeys were evaluated at 1 day, 7 days, 2 weeks, 4 weeks, 8 weeks, and 12 weeks post-MCAO using the non-human primate stroke scale (NHPSS) as previously described, which is analogous to the National Institutes of Health Stroke Scale utilized in clinical stroke patients [21]. A score of 0 corresponds to normal neurological function and 41 to severe bilateral neurological deficits. Neurological deficits in rats were assessed 24 h after MCAO using the Garcia test as previously described [22]. The Garcia test evaluates spontaneous activity, forepaw outstretching, symmetry in the movement of the four limbs, body proprioception, climbing, and the response to vibrissa touch. A score of 18 corresponds to normal neurological function, and 0 corresponds to severe neurological deficits.

### Cognition assessment in cynomolgus monkeys and rats

The cognitive function of cynomolgus monkeys was evaluated using the delayed response test (DRT) 12 weeks post-stroke, as previously described [23]. Briefly, the DRT was conducted in a modified Wisconsin General Test Apparatus (WGTA). Cynomolgus monkeys were shown which well the bait was put in. Then, the opaque Plexiglas cover slid down. Following a delay, the cover slid up and monkeys were required to retrieve the hidden food from one of the three wells **(Fig. S1)**. The bait locations were randomized across the left, right, and center wells over 10 task trials. Each monkey underwent training pre-surgery and was tested 12 weeks post-sham or MCAO surgery. Subsequently, the first-time success rate was calculated.

The cognitive function of SD rats was assessed using the Morris water maze and novel object recognition (NOR) as previously described [24,25]. Spatial acquisition trials were conducted for six consecutive days (from 22 to 27 days post-surgery), followed by a probe trial on the 28th day post-surgery. During the probe trial, swimming duration was fixed at 60 s for all rats. The dwell time in the target quadrant was recorded and reported as absolute seconds and as a percentage of the 60-s trial. NOR testing was conducted in a square open-field arena (50 × 50 × 40 cm). Animals underwent habituation at day 26 post-stroke, the familiarization phase (two identical objects) at day 27 post-stroke, and test phase (one familiar object and one novel object) at day 28 post-stroke. The discrimination index (DI) was calculated as: DI = (T_novel_ − T_familiar_)/(T_novel_ + T_familiar_).

### Tissue preparation

All cynomolgus monkeys and rats were euthanized at 12 and 4 weeks after MCAO, respectively. The animals were deeply anesthetized with pentobarbital (50 mg/kg, intramuscular injection). The tibialis anterior (TA) or quadriceps femoris (QFM) muscle on both sides was promptly excised and sectioned into small pieces. Some pieces were longitudinally oriented, embedded in OCT, and flash-frozen in liquid nitrogen-cooled isopentane for histological analyses. The remaining fragments were snap-frozen in liquid nitrogen for protein extraction. Following muscle removal, the animals underwent transcardial perfusion with saline. For western blot analysis, the brains were extracted, and the bilateral hippocampi were dissected and promptly snap-frozen in liquid nitrogen. For histological analysis, animals were perfused with 4 % paraformaldehyde. The brains were then extracted, fixed in 4 % paraformaldehyde, and embedded in paraffin. Coronal sections including hippocampus were used.

### Immunofluorescence/Immunohistochemistry

TA and QFM muscles embedded in OCT were cryosectioned into 10 μm thickness, while brains embedded in paraffin were sectioned into 3 μm thickness. The frozen or deparaffinized sections were incubated sequentially in 0.2 % TX-100 and 0.3 %–3% H_2_O_2_ for 20 min at room temperature, followed by blocking with 5 % bovine serum albumin (BSA, V900933, Sigma) for 1 h. Subsequently, the sections were incubated overnight at 4 °C with primary antibodies, including anti-laminin (ab11575, Abcam, Cambridge, UK, 1:100), anti-FNDC5 (ab174833, Abcam, Cambridge, UK, 1:200), anti-BDNF (ab108319, Abcam, Cambridge, UK, 1:200), anti-doublecortin (DCX, ab207175, Abcam, Cambridge, UK, 1:200) and anti-iba1 (ab178846, Abcam, Cambridge, UK, 1:400). For immunofluorescence, the sections were then treated with secondary antibodies (Goat Anti-Rabbit/Mouse Alexa Fluor 488/594) and mounted in Fluoroshield™ with DAPI (F6057, Sigma). In the case of immunohistochemistry, peroxidase-labeled rabbit/mouse secondary antibodies were used, followed by DAB staining with an IHC kit (XYM-Dako-K5007). Nuclei were counterstained with hematoxylin (BA4021A, Baso), dehydrated, and mounted on neutral balsam.

All images were captured using a Nikon microscope and analyzed using the ImageJ software. Over 150 muscle fibers per animal were randomly selected to measure the average fiber CSA. Two sections of the hippocampal CA1 or dentate gyrus were utilized (3–5 fields per section) to quantify the expression level of BDNF or the number of DCX^+^ cells by investigators who were blinded to the animals’ information.

### Nissl staining

For rat brain sections, Nissl staining was performed with Nissl staining solution (Beyotime, C0117). Images were captured using a slide scanner (KFBIO, KF-PRO-020) to assess infarction.

### Western blot analysis

Protein was extracted by homogenization in RIPA (89900, Thermo Fisher) and PMSF (78430, Thermo Fisher). The protein concentration was determined using BCA (23227, Thermo Fisher). Equal amounts of protein were separated on SDS-PAGE gels (7.5 %, 10 %, or 12.5 %) in Tris-glycine-SDS buffer and transferred to a polyvinylidene difluoride membrane (PVDF, ISEQ00010, IPVH00010, Millipore). The PVDF membranes were incubated in 5 % nonfat milk for 1 h at room temperature and then with primary antibodies at 4°C overnight. Primary antibodies included anti-myosin (skeletal, fast, M4276, Sigma, 1:2000), anti-PGC-1α (ab188102, Abcam, 1:2000), anti-FNDC5 (ab174833, Abcam, 1:2000), anti-BDNF (ab108319, Abcam, 1:2000), anti-Doublecortin (ab207175, Abcam, 1:2000), and anti-PSD-95 (ab238135, Abcam, 1:2000). Subsequently, the membranes were incubated with HRP-linked secondary antibody and visualized using an Amersham Imager 600 with Immobilon Western HRP Substrate (WBKLS0500, Millipore). Band intensities were quantified using ImageJ software.

### Enzyme-linked immunosorbent assay

Blood samples from the monkeys were collected before, 4 weeks after, and 12 weeks after the operation, and blood samples from SD rats were collected 28 days after the operation. The samples were centrifuged at 3000 × rpm at 4 °C for 10 min to obtain the serum, which was stored at − 80 °C until further analysis. Serum irisin levels in cynomolgus monkeys and SD rats were quantified using a commercial Irisin Competitive ELISA kit (AG-45A-0046YEK-KI01, Adipogen) and a Rat Irisin ELISA kit (CSB-EL008770RA, CUSABIO) following the manufacturer’s protocol. The linear range of the standard curve was 0.001 μg/mL to 5 μg/mL (AG-45A-0046YEK-KI01, Adipogen) or 0.625 ng/mL to 40 ng/mL (CSB-EL008770RA, CUSABIO), with intra- and inter-assay coefficients of variation being 4.8 % to 8.2 % and 8.0 % to 9.7 %, respectively (AG-45A-0046YEK-KI01, Adipogen) or approximately 8 % to 10 %, respectively (CSB-EL008770RA, CUSABIO).

### Statistical analysis

Statistical analyses were conducted using IBM SPSS Statistics version 25 (IBM Corp., Armonk, NY, USA). The normality of distribution was assessed with the Kolmogorov-Smirnov test. Normally distributed data were reported as mean ± standard deviation, while non-normally distributed data were presented as median and interquartile range, respectively. Expression levels among different groups were compared using an unpaired *t*-test or One-Way ANOVA, followed by Bonferroni’s post hoc test for normally distributed data and the Mann–Whitney test for non-normally distributed data. Temporal changes in serum irisin levels were assessed using two-way repeated measures ANOVA (group × time). Mid-thigh skeletal muscle CSA and fat fraction comparisons between the paretic and non-paretic sides were conducted using a paired *t*-test. The associations between serum irisin levels and muscle CSA, post-stroke cognition, and hippocampus BDNF were explored through linear regression analysis. The significance level was set at *α* = 0.05.

## Results

### Correlation of serum irisin levels with muscle atrophy and post-stroke cognitive impairment in stroke patients

We enrolled 24 first-time stroke survivors 3–6 months after the onset, and 24 healthy controls matched for age and vascular risk factors. Twenty-two stroke patients underwent thigh MRI scans, cognitive evaluation, and blood sampling, while the remaining two underwent blood sampling alone. All patients had normal swallowing function at discharge, which minimized the potential risk of malnutrition associated with post-stroke dysphagia. In all included patients, albumin levels were > 35 g/L at baseline (during hospitalization) and follow-up (3–6 months post-stroke), and a paired *t*-test showed no significant change from baseline (41.2 g/L ± 4.2 g/L) to follow-up (42.2 g/L ± 2.6 g/L), suggesting that chronic malnutrition was unlikely in the included patients **(Table S1)**. Among the 22 patients with cerebral infarction, 18 lesions (81.8 %) involved the white matter, 1 lesion (4.6 %) involved the cortex, and 3 lesions (13.6 %) involved both the cortex and white matter. The median infarct volume was 6.02 ml (IQR 2.18–10.29 ml). The clinical profiles of the patients are summarized in **Table S1**.

**Fig. 1:**
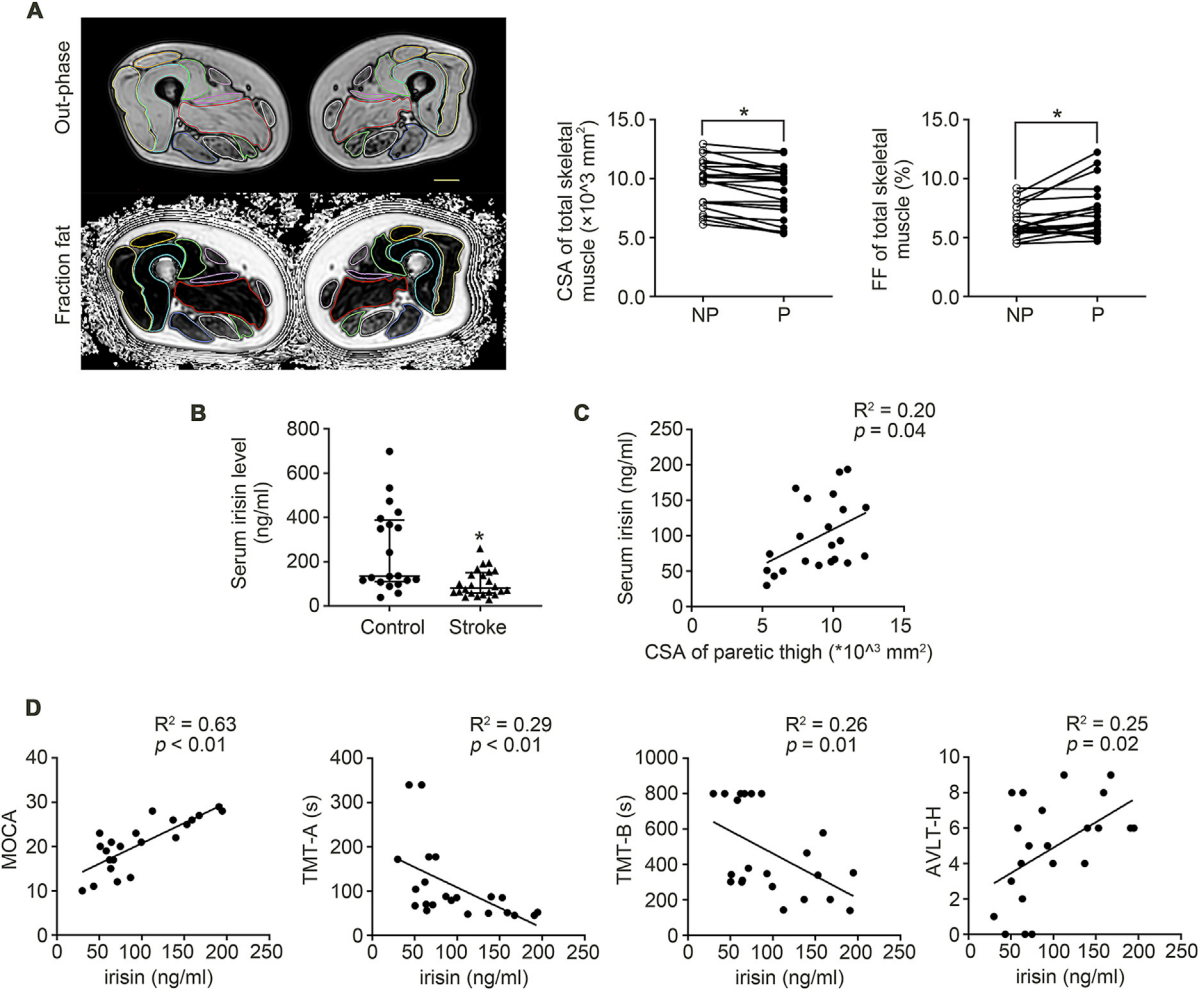
Correlation between serum irisin level and post-stroke cognitive impairment. (A) Representative images of mid-thigh mDIXON-QUANT and analysis of CSA and fat fraction of skeletal muscle between hemiplegic and non-hemiplegic thigh in stroke patients (*n* = 22). Top left: Out-phase image for region of interest (ROI) segmentation; bottom left: Fraction fat image for CSA and fat fraction quantification. ROIs are manually segmented on the out-phase image at the mid-thigh level and subsequently applied to the fat fraction image for quantification of CSA and fat fraction. Scale bar, 20 cm. (B) Analysis of serum irisin level between stroke patients and healthy controls (*n* = 20 for healthy control, *n* = 24 for stroke group). (C) Correlation between total CSA of paretic thigh and serum irisin level in stroke patients (*n* = 22). (D) Correlation between serum irisin level and global cognition (MoCA), executive function (TMT-A, TMT-B), and memory (ALTH) in stroke patients (*n* = 22). CSA, cross-sectional area; FF, fraction fat; NP, nonparetic; P, paretic. Serum irisin (B) is presented as median and interquartile range. *p < 0.05 versus nonparetic thigh (A) or healthy control (B), Paired *t*-test (A), Mann–Whitney test (B), Simple linear regression (C, D).

In stroke patients, thigh mDIXON-QUANT MRI was used to measure the CSA and FF of skeletal muscle **(**Fig. 1**A)**. The paretic thigh exhibited a significant decrease in total skeletal muscle CSA compared to the nonparetic side (8935 mm^2^ ± 2214 mm^2^ vs. 9564 mm^2^ ± 2039 mm^2^; *p* < 0.05) and an increase in FF (7.17 % ± 2.15 % vs. 6.31 % ± 1.35 %; *p* < 0.05). Further analysis of single skeletal muscle indicated that vastus lateralis, vastus medialis, vastus intermedius, rectus femoris, adductor magnus and biceps femoris exhibited significantly reduced CSA and increased FF (all *p* < 0.05, **Fig. S2 and S3**). Serum irisin levels were significantly lower in stroke patients compared to healthy controls (median (IQR) 80.7 ng/mL (59.1 ng/mL, 149.7 ng/mL) versus 134.7 ng/mL (110.0 ng/mL, 388.3 ng/mL); *p* < 0.05, Fig. 1**B**). In stroke patients, the total CSA of the paretic thigh showed a positive correlation with serum irisin levels (*R*^2^ = 0.20, *p* < 0.05; Fig. 1**C**). Additionally, higher serum irisin levels were associated with enhanced global cognition (MoCA, *R*^2^ = 0.63, *p* < 0.05), executive function (TMT-A, *R*^2^ = 0.29, *p* < 0.05; TMT-B, *R*^2^ = 0.26, *p* < 0.05), and memory (AVLT-H, *R*^2^ = 0.25, *p* < 0.05; Fig. 1**D**). To validate the robustness of the association between serum irisin levels and MoCA, additional analyses using linear regression and bootstrapping (1,000 resamples) were performed. The results remained consistent, and the bootstrapped 95 % confidence interval for the MoCA association did not cross zero (B = 0.092, 95 % CI 0.059–0.125, β = 0.793, R^2^ = 0.63; *p* < 0.05). After adjustment for infarct volume, serum irisin levels remained positively correlated with global cognition (MoCA, B = 0.089, 95 % CI 0.056–0.123; *p* < 0.05), executive function (TMT-A, B = −0.813, 95 % CI −1.435 to −0.191, *p* < 0.05; TMT-B, B = −2.435, 95 % CI −4.478 to −0.391, *p* < 0.05), and memory (AVLT-H, B = 0.028, 95 % CI 0.004–0.052; *p* < 0.05, **Table S2**).

### Cognitive impairment and hemiplegic muscle atrophy post-MCAO in cynomolgus monkeys

**Fig. 2:**
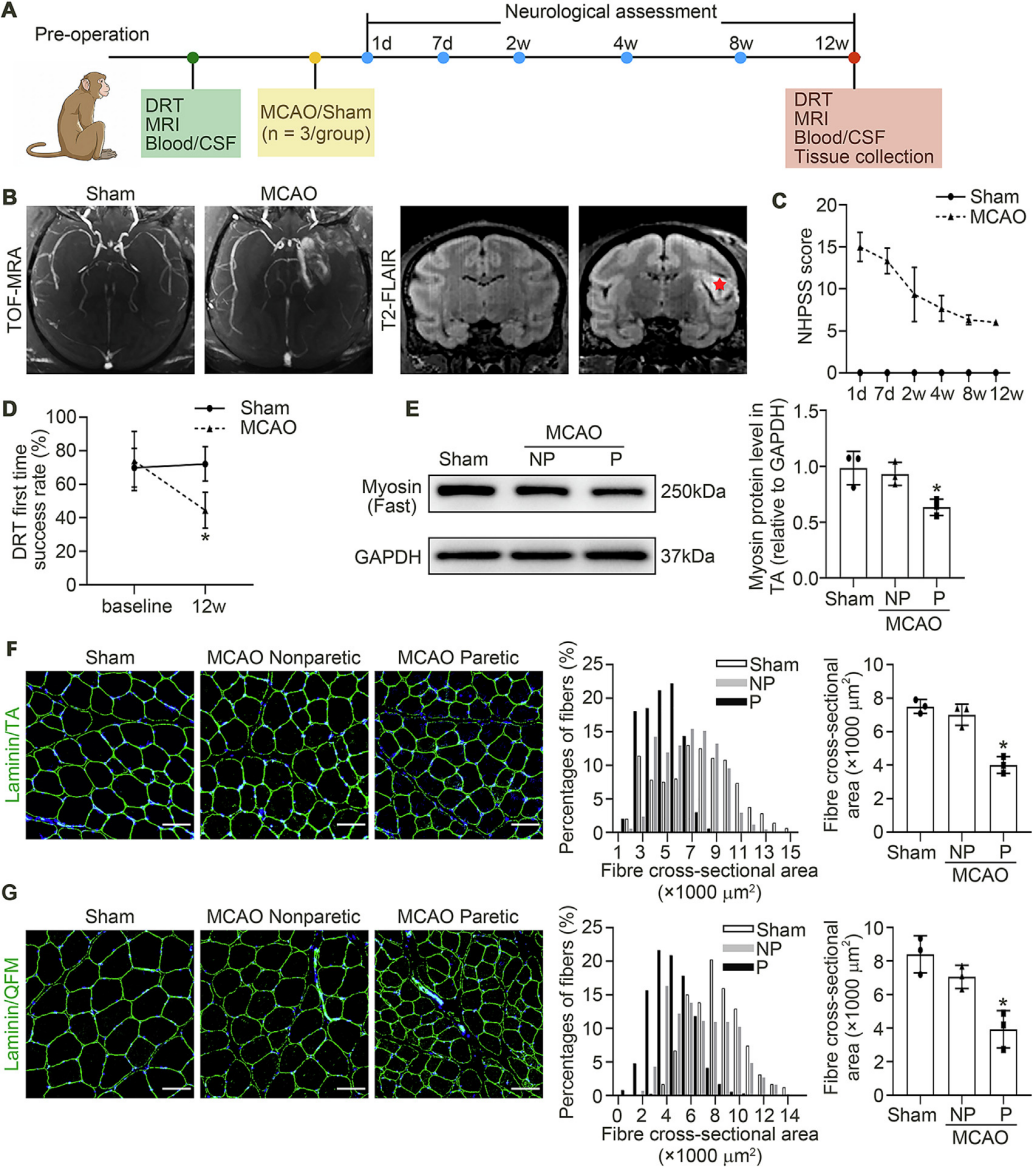
Neurological deficits and paretic muscle atrophy 12 weeks after stroke in cynomolgus monkeys. (A) Outline of the cynomolgus monkey experimental design. (B) Successful MCAO verified by TOF-MRA and infarct lesion shown by T2-FLAIR 12 weeks after the operation. The star represents the infarct lesion. (C) Neurological deficits evaluated by NHPSS scaling post-MCAO or sham operation in cynomolgus monkeys (*n* = 3 per group). (D) Post-stroke cognition evaluated by DRT 12 weeks after MCAO or sham operation in cynomolgus monkeys (*n* = 3 per group). (E) Representative western blot bands and quantitative analysis of fast myosin protein expression in TA muscle 12 weeks after MCAO or sham operation (*n* = 3 per group). (F, G) Representative images of laminin immunofluorescence staining of TA (F) and QFM muscle (G) 12 weeks post-MCAO or sham operation and fiber size distribution and fiber mean CSA of TA (F) and QFM muscle (G) calculated using laminin immunostaining. More than 200 fibers are randomly used for CSA quantification per animal (*n* = 3 per group). Scale bar, 100 μm. CSA, cross-sectional area; DRT, delayed response test; NP, nonparetic; P, paretic; QFM, quadriceps femoris; TA, tibialis anterior. All data are presented as mean ± SD. *p < 0.05 versus MCAO baseline (D) or sham (E, F, G), Two-way ANOVA (D), One-way ANOVA (E, F, G) followed by Bonferroni’s post hoc.

Nonhuman primates offer a highly translatable model for neuroscience studies. We then examined whether stroke-induced skeletal muscle loss impacts the brain through the muscle–brain axis FNDC5/irisin/BDNF in cynomolgus monkeys. The experimental setup for cynomolgus monkeys was illustrated in Fig. 2**A**. Successful induction of MCAO was confirmed by TOF-MRA, with T2 FLAIR revealing infarct lesions predominantly in the parietal cortex and striatum while sparing the hippocampus (Fig. 2**B**). Twelve weeks post-MCAO, the cynomolgus monkeys exhibited persistent hemiparesis and decreased voluntary movement in both upper and lower extremities, with an average NHPSS score of 6 **(**Fig. 2**C)**. The sham-operated animals consistently scored 0 on the NHPSS throughout the study. The DRT was performed to evaluate post-stroke cognition (**Fig. S1**). The stroke-afflicted monkeys exhibited a significantly lower first-time success rate in the DRT at 12 weeks compared to baseline (44.5 % ± 10.4 % vs. 73.9 % ± 17.7 %; *p* < 0.05, Fig. 2**D**).

Western blot analysis revealed a significant reduction in myosin protein levels in the affected limb compared to the sham group (*p* < 0.05, Fig. 2**E**). Muscle morphometry of the TA and QFM was assessed using laminin immunostaining 12 weeks after MCAO. In comparison to the sham group, the fiber size distribution of the paretic TA and QFM in the MCAO group shifted towards smaller fibers. The average fiber cross-sectional area (CSA) decreased by approximately 40 % in the paretic TA (4017 μm^2^ ± 495 μm^2^ vs. 7507 μm^2^ ± 412 μm^2^; *p* < 0.05, Fig. 2**F**), and reduced by approximately 56 % in the paretic QFM (3932 μm^2^ ± 1104 μm^2^ vs. 8397 μm^2^ ± 1098 μm^2^; *p* < 0.05, Fig. 2**G**).

### Downregulation of the FNDC5/irisin/BDNF axis post-MCAO in cynomolgus monkeys

**Fig. 3:**
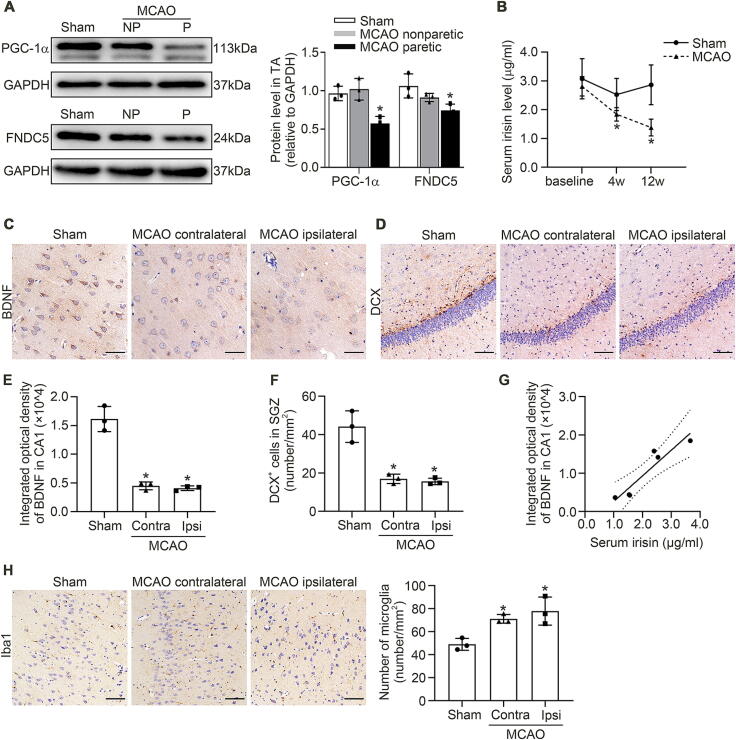
PGC-1α/FNDC5/irisin/BDNF axis downregulation 12 weeks post-stroke in cynomolgus monkeys. (A) Representative Western blot bands and quantitative analysis of PGC-1α/FNDC5 protein expression levels in TA muscle 12 weeks post-MCAO or sham operation (n = 3 per group). (B) Temporal changes in serum irisin levels in the MCAO or sham group (n = 3 per group). (C, E) Representative images of BDNF immunohistochemistry staining in bilateral hippocampus CA1 12 weeks post-MCAO or sham operation, and quantitative analysis of BDNF integrated optical density (n = 3 per group). Scale bar, 50 μm. (D, F) Representative images of DCX immunohistochemistry staining in bilateral hippocampus SGZ 12 weeks post-MCAO or sham operation, and quantitative analysis of DCX^+^ cell number (n = 3 per group). Scale bar, 100 μm. Two different sections and 3–5 fields in each section are used for optical density or DCX^+^ cell number quantification per animal. (G) Linear regression analysis between serum irisin and hippocampal BDNF. (H) Representative images of Iba1 immunohistochemistry staining in bilateral hippocampus 12 weeks post-MCAO or sham operation, and quantitative analysis of microglial count (n = 3 per group). Scale bar, 100 μm. Contra, contralateral to the infarct; Ipsi, ipsilateral to the infarct; NP, nonparetic; P, paretic; SGZ, subgranular zone. All data are presented as mean ± SD. *p < 0.05 versus sham (A, E, F, H) or baseline (B), Simple linear regression analysis (G), One-way ANOVA (A, E, F, H), Two-way ANOVA (B), followed by Bonferroni’s post hoc.

Western blotting and immunofluorescence staining revealed a significant decrease in FNDC5 protein expression in the paretic TA 12 weeks after MCAO in cynomolgus monkeys compared to the sham group (*p* < 0.05, Fig. 3**A and Fig. S4**). PGC-1α, an upstream regulator of FNDC5 [14], was also notably reduced in the paretic TA compared to the sham (*p* < 0.05, Fig. 3**A**). Monkeys exhibited significantly lower serum irisin levels at 4 weeks (1.8 μg/ml ± 0.2 μg/ml) and 12 weeks (1.4 μg/mL ± 0.3 μg/mL) after MCAO compared to baseline (2.8 μg/ml ± 0.3 μg/ml; all *p* < 0.05, Fig. 3**B**). In contrast, the serum irisin levels remained constant in the sham group **(**Fig. 3**B**). Reduced CSF irisin levels were also observed at 4 weeks (0.63 μg/mL ± 0.17 μg/mL) and 12 weeks (0.48 μg/mL ± 0.12 μg/mL) post-MCAO compared to baseline (1.19 μg/ml ± 0.04 μg/ml; all *p* < 0.05, **Fig.S5**).

Along with decreased peripheral PGC-1α/FNDC5/irisin levels, immunohistochemistry showed significantly reduced BDNF expression in both hippocampus CA1 regions 12 weeks post-MCAO compared to the sham group (*p* < 0.05, Fig. 3**C and E**). Importantly, in our primate stroke model, the hippocampi were distal from the primary infarct **(**Fig. 2**B)**, suggesting that the decline in BDNF expression might be due to a disrupted muscle-brain axis rather than direct ischemic damage. BDNF expression correlates with neurogenesis in the subgranular zone (SGZ) of the hippocampus [26]. We observed a significant decrease in the number of immature neurons, identified by DCX, in both the ipsilateral (16/mm^2^ ± 2/mm^2^) and contralateral (17/mm^2^ ± 2/mm^2^) SGZ regions 12 weeks post-MCAO compared to the sham group (44/mm^2^ ± 8/mm^2^; all *p* < 0.05, Fig. 3**D and F**). Moreover, linear regression analysis revealed a positive correlation between serum irisin levels and hippocampal BDNF (R^2^ = 0.88, *p* < 0.05, Fig. 3**G**). Iba1 immunostaining demonstrated a significant increase in the microglial count in both ipsilateral (78/mm^2^ ± 12/mm^2^) and contralateral hippocampus (71/mm^2^ ± 4/mm^2^) 12 weeks post-MCAO compared with the sham group (49/mm^2^ ± 5/mm^2^; *p* < 0.05, Fig. 3**H**).

### Downregulation of the FNDC5/irisin/BDNF axis and cognitive impairment post-MCAO in rats

Given that FNDC5/Irisin is highly conserved across species, we investigated whether rats exhibited impaired PGC-1α/FNDC5/irisin/BDNF axis post-stroke, similar to monkeys **(Fig. S6A)**. Infarcts were identified by Nissl staining and were excluded from the hippocampus **(Fig. S6B)**. Rats displayed hemiparesis following MCAO, with an average score of 10 (10.1 ± 1.6 score) on the Garcia test one day after MCAO **(Fig. S6C**). PSCI in rats was evaluated using the Morris water maze 28 days after MCAO. Compared to the sham group, MCAO rats exhibited poorer spatial memory retention in the probe trial, spending significantly less time in the targeted quadrant (12.7 s ± 3.7 s vs. 31.3 s ± 4.1 s; *p* < 0.05, **Fig. S6D**). Similar to stroke patients and monkeys, the CSA of the paretic TA was significantly smaller in stroke rats compared to the nonparetic side or sham group (1460 μm^2^ ± 263 μm^2^ vs. 2764 μm^2^ ± 684 μm^2^ vs. 3152 μm^2^ ± 622 μm^2^; *p* < 0.05, **Fig. S6E**), along with reduced myosin protein expression (*p* < 0.05, **Fig. S6F**). Furthermore, the PGC-1α/FNDC5/Irisin/BDNF axis was significantly downregulated 28 days post-MCAO in rats compared to the sham group (all *p* < 0.05, **Fig. S7A-C**). The decreased BDNF expression was accompanied by reduced PSD95 and DCX protein expression in the bilateral hippocampi relative to the sham group (all *p* < 0.05, **Fig. S7C**). MCAO rats exhibited significantly fewer DCX^+^ cells in both the ipsilateral (22/mm^2^ ± 3/mm^2^) and contralateral (21/mm^2^ ± 4/mm^2^) SGZ compared to the sham group (66/mm^2^ ± 5/mm^2^; all *p* < 0.05, **Fig. S7D**). Moreover, microglial count in the ipsilateral (98/mm^2^ ± 7/mm^2^) and contralateral hippocampus (88/mm^2^ ± 5/mm^2^) was significantly increased when compared with the sham group (58/mm^2^ ± 2/mm^2^; *p* < 0.05, **Fig. S7E**).

### Exercise-induced upregulation of the FNDC5/irisin/BDNF axis and cognitive improvement post-MCAO in rats

**Fig. 4:**
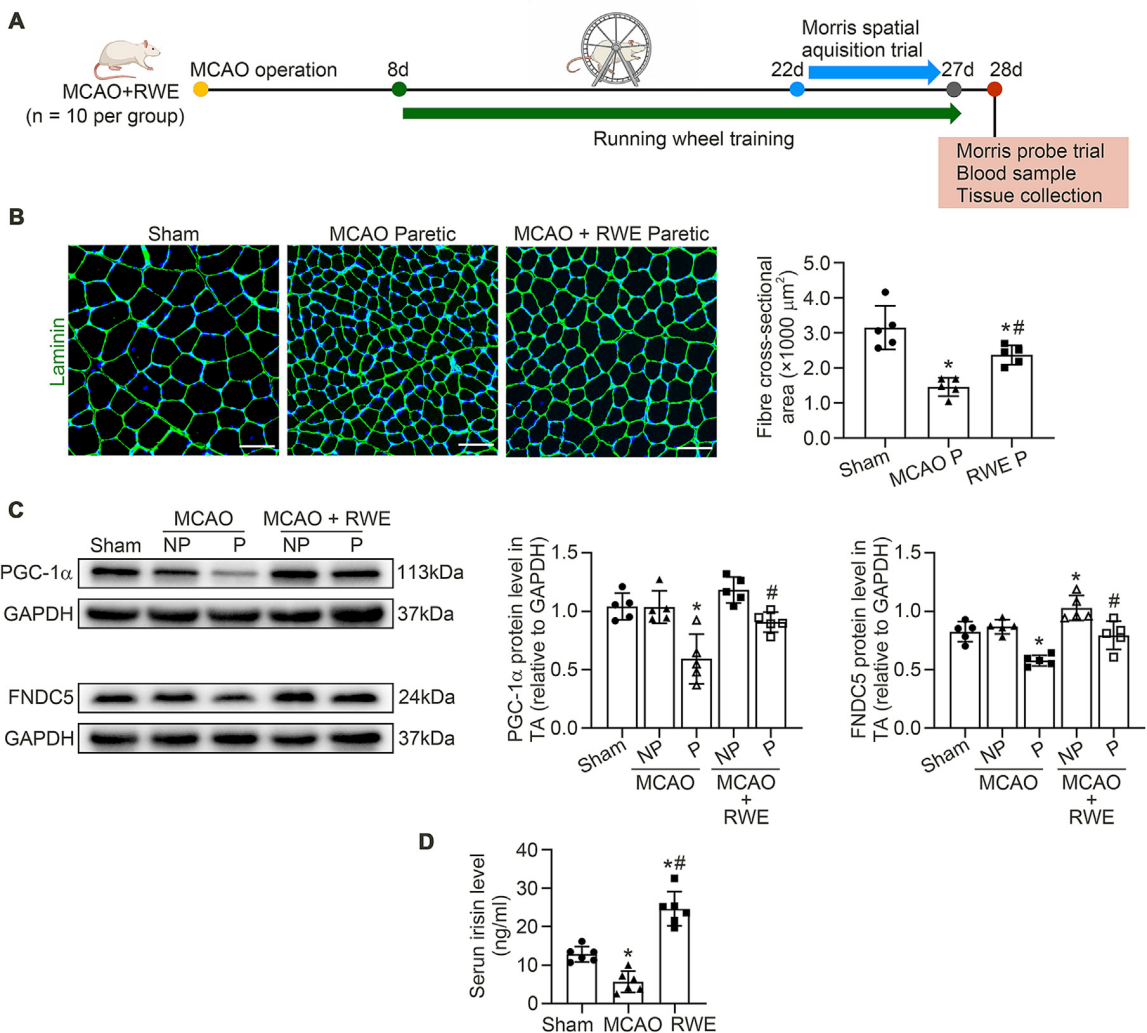
Effects of exercise on paretic muscle atrophy and PGC-1α/FNDC5/irisin expression 28 days after stroke in rats. (A) Outline of the running wheel exercise design. (B) Representative images of laminin immunofluorescence staining of TA muscle 28 days post-operation and fiber mean CSA of TA muscle calculated by laminin immunostaining. More than 200 fibers are randomly used for CSA quantification per animal (*n* = 5 per group). Scale bar, 100 μm. (C) Representative Western blot bands and quantitative analysis of PGC1α/FNDC5 protein expression level in TA muscle of sham, MCAO, and MCAO + Exercise groups 28 days after operation (*n* = 5 per group). (D) Quantitative analysis of serum irisin level of sham, MCAO, and MCAO + Exercise groups 28 days post-operation (*n* = 6 per group). NP, nonparetic; P, paretic; RWE, running wheel exercise; TA, tibialis anterior. All data are presented as mean ± SD. *p < 0.05 versus sham, #*p* < 0.05 versus MCAO (B, D) group or corresponding side of MCAO group (C), One-way ANOVA followed by Bonferroni’s post hoc.

Irisin is released from skeletal muscles in response to physical exercise and is clinically practical and feasible. We investigated whether physical exercise enhances the FNDC5/irisin/BDNF axis post-MCAO in rats and ameliorates PSCI. Rats underwent running wheel exercises from days 8 to 27 post-MCAO **(**Fig. 4**A)**. Twenty-eight days post-stroke, the fiber size of the paretic TA muscle was significantly larger in the MCAO + Exercise group than in the MCAO group (2375 μm^2^ ± 274 μm^2^ vs. 1460 μm^2^ ± 263 μm^2^; *p* < 0.05, Fig. 4**B**). Physical exercise upregulated PGC-1a and FNDC5 protein levels in the paretic TA compared to the MCAO group (all *p* < 0.05). Additionally, FNDC5 protein significantly increased in the nonparetic TA of the MCAO + Exercise group compared to the sham group (*p* < 0.05, Fig. 4**C**). Notably, the MCAO + Exercise group exhibited higher serum irisin levels (24.7 ng/mL ± 4.4 ng/mL) than the MCAO (5.6 ng/mL ± 2.8 ng/mL) and sham groups (12.8 ng/mL ± 2.0 ng/mL; all *p* < 0.05, Fig. 4**D**).

**Fig. 5:**
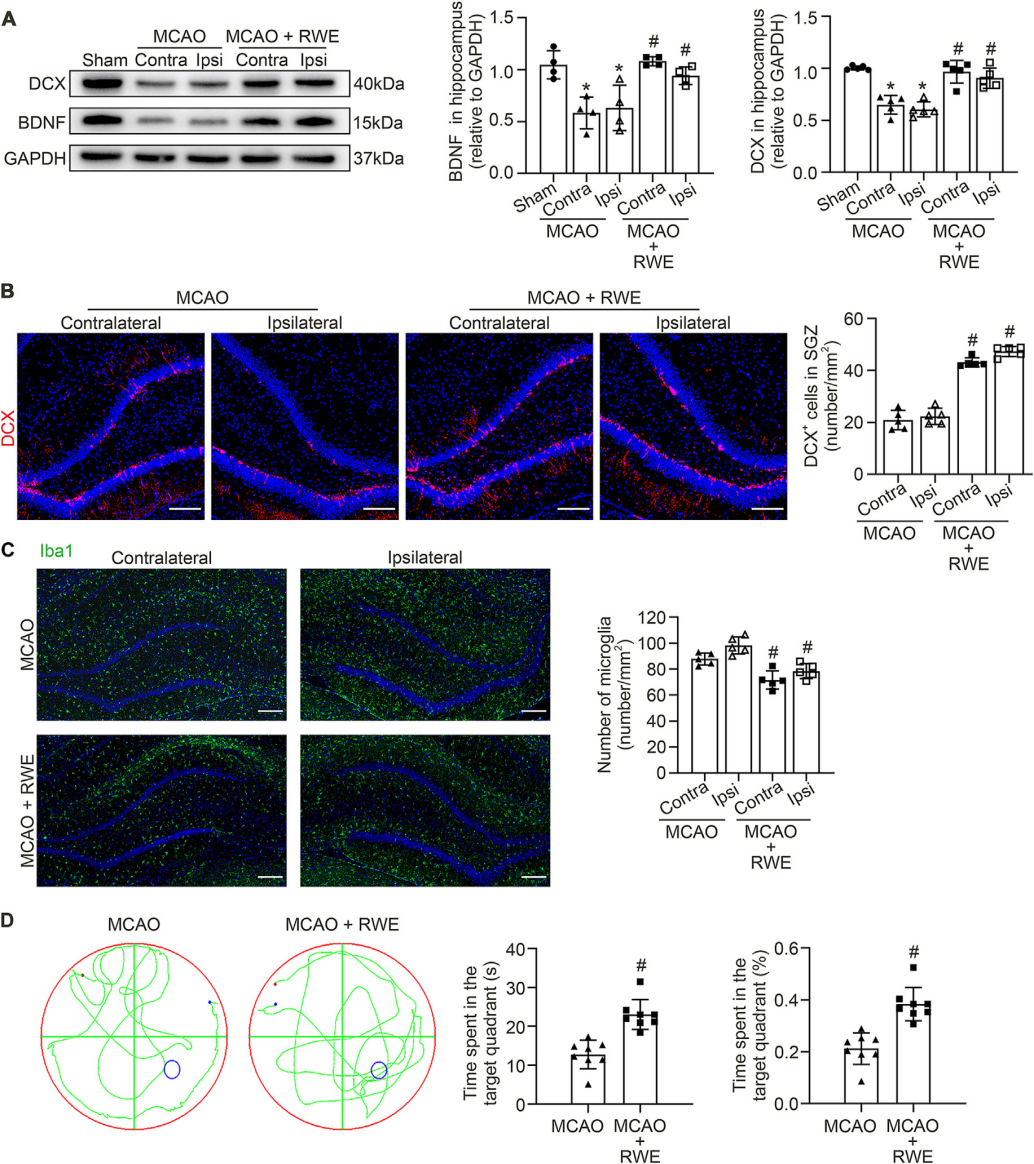
Exercise-induced increase of irisin enhancing BDNF expression and promoting immature neuron formation in the hippocampus following stroke in rats. (A) Representative Western blot bands and quantitative analysis of BDNF and DCX protein expression levels in bilateral hippocampi of sham, MCAO, and MCAO + Exercise groups 28 days after operation (*n* = 4–5 per group). (B) Representative images of DCX immunostaining in bilateral hippocampi SGZ of MCAO and MCAO + Exercise groups 28 days post-operation, and quantitative analysis of DCX^+^ cell number in bilateral hippocampi SGZ (*n* = 5 per group). Scale bar, 200 μm. Three different sections and three fields in each section are used for DCX^+^ cell number quantification per animal. (C) Representative images of Iba1 immunostaining in bilateral hippocampi of MCAO and MCAO + Exercise groups 28 days post-operation, and quantitative analysis of microglial count in bilateral hippocampi (*n* = 5 per group). Scale bar, 250 μm. (D) Representative probe trial swimming traces and quantitative analysis of time spent in the target quadrant 28 days post-operation in MCAO and MCAO + Exercise groups (*n* = 8 per group). Contra, contralateral to the infarct; Ipsi, ipsilateral to the infarct; RWE, running wheel exercise, SGZ, subgranular zone. All data are presented as mean ± SD. *p < 0.05 versus sham, #*p* < 0.05 versus corresponding side of MCAO group, One-way ANOVA followed by Bonferroni’s post hoc (A, B, C), Unpaired *t*-test (D).

Exercise-induced irisin release significantly increased BDNF and DCX expression in the bilateral hippocampi compared with the MCAO group (all *p* < 0.05, Fig. 5**A**). The MCAO + Exercise group had significantly more DCX^+^ neurons in the bilateral hippocampi (ipsilateral, 43/mm^2^ ± 2/mm^2^; contralateral, 47/mm^2^ ± 2/mm^2^) when compared with the MCAO group (ipsilateral, 22/mm^2^ ± 3/mm^2^; contralateral, 21/mm^2^ ± 4/mm^2^; all *p* < 0.05, Fig. 5**B**). Iba1 immunostaining demonstrated a significantly reduced number of microglia in the bilateral hippocampi of the MCAO + Exercise group (ipsilateral, 78/mm^2^ ± 6/mm^2^; contralateral, 72/mm^2^ ± 7/mm^2^) compared with the MCAO group (ipsilateral, 98/mm^2^ ± 7/mm^2^; contralateral, 89/mm^2^ ± 5/mm^2^; *p* < 0.05, Fig. 5**C**). Twenty-eight days after MCAO, Morris water maze demonstrated that the MCAO + Exercise group spent significantly more time in the targeted quadrant than the MCAO group (23.0 s ± 3.8 s vs. 12.7 s ± 3.7 s; *p* < 0.05, Fig. 5**D**).

**Fig. 6:**
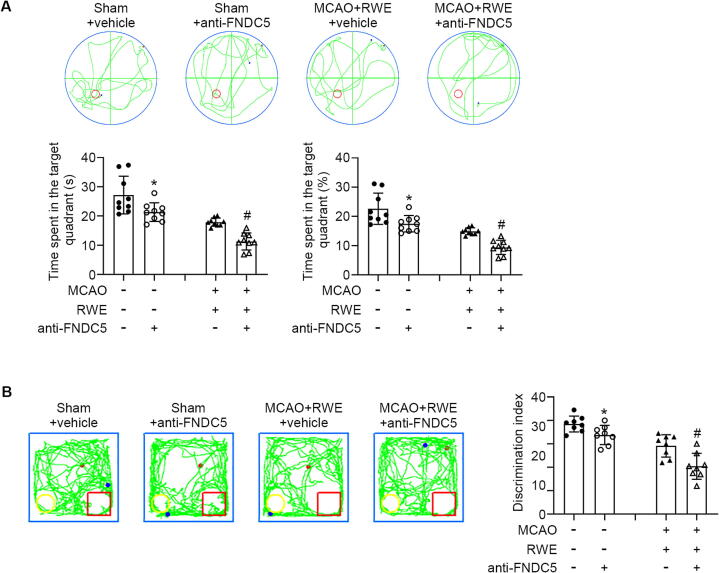
Neutralizing anti-FNDC5 antibody impedes cognitive recovery after stroke in rats. (A) Representative probe trial swimming traces and quantitative analysis of time spent in the target quadrant 28 days post-operation in sham + vehicle, sham + anti-FNDC5, MCAO + Exercise + vehicle, MCAO + Exercise + anti-FNDC5 groups (*n* = 9 per group). (B) Representative NOR tracking traces and quantitative analysis of discrimination index 28 days post-operation in sham + vehicle, sham + anti-FNDC5, MCAO + Exercise + vehicle, MCAO + Exercise + anti-FNDC5 groups (*n* = 8 per group). RWE, running wheel exercise. All data are presented as mean ± SD. *p < 0.05 versus sham + vehicle, #*p* < 0.05 versus MCAO + Exercise + vehicle group, Unpaired *t*-test.

To verify that muscle-derived FNDC5/irisin mediated the cognitive benefits of exercise post-stroke, rats were intravenously injected with an anti-FNDC5 antibody via tail vein. Administration of the anti-FNDC5 antibody impaired cognitive performance under normal conditions. Compared with the sham + vehicle group, the sham + anti-FNDC5 group spent significantly less time in the targeted quadrant in the Morris water maze (21.3 s ± 3.2 s vs. 27.1 s ± 6.4 s; *p* < 0.05, Fig. 6**A**), and had significantly lower discrimination index in the NOR test (0.68 ± 0.08 vs. 0.77 ± 0.07; *p* < 0.05, Fig. 6**B**). In stroke rats, anti-FNDC5 blocked the protective effects of exercise on post-stroke cognition. Rats in the MCAO + Exercise + anti-FNDC5 group spent significantly less time in the targeted quadrant in the Morris water maze (11.3 s ± 2.9 s vs. 17.9 s ± 1.4 s; *p* < 0.05, Fig. 6**A**), and showed a significantly lower discrimination index in the NOR test (0.41 ± 0.11 vs. 0.58 ± 0.10; *p* < 0.05, Fig. 6**B**).

### Protective effects of peripheral FNDC5/irisin overexpression on hippocampal BDNF and PSCI in rats

**Fig. 7:**
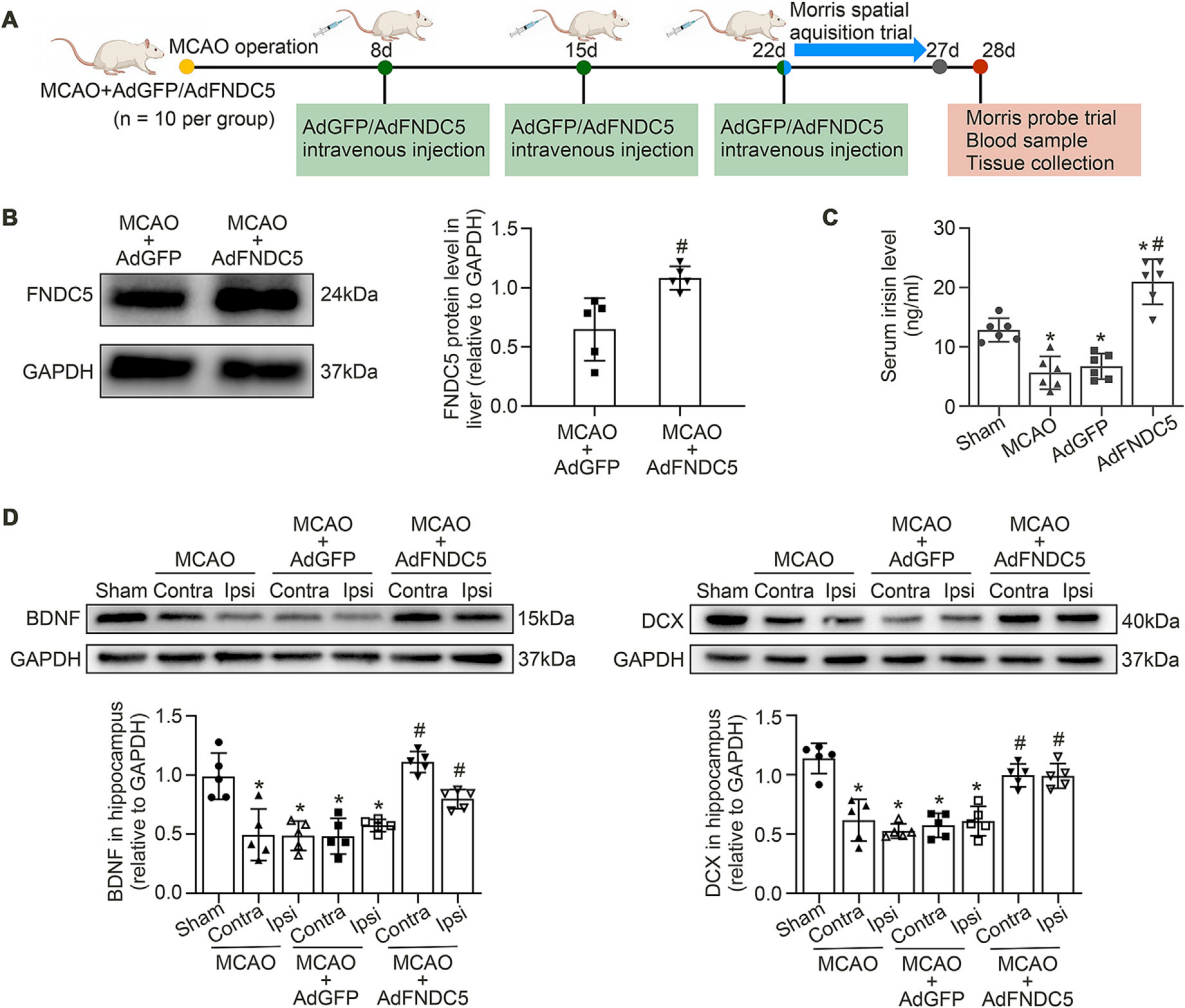
Peripheral FNDC5/irisin overexpression promoting BDNF expression in hippocampus after stroke in rats. (A) Schematic of intravenous injection of GFP- or FNDC5-expressing adenoviral particles into the tail vein following stroke in rats. (B) Representative Western blot bands and quantitative analysis of FNDC5 protein expression level in the liver of MCAO + AdGFP and MCAO + AdFNDC5 groups 28 days after operation (*n* = 5 per group). (C) Quantitative analysis of serum irisin levels in sham, MCAO, MCAO + AdGFP, and MCAO + AdFNDC5 groups 28 days post-operation (*n* = 6 per group). (D) Representative Western blot bands and quantitative analysis of BDNF and DCX protein expression level in the bilateral hippocampi of sham, MCAO, MCAO + AdGFP, and MCAO + AdFNDC5 28 days post-operation (*n* = 5 per group). Contra, contralateral to the infarct; Ipsi, ipsilateral to the infarct. All data are presented as mean ± SD. *p < 0.05 versus sham, #*p* < 0.05 versus MCAO + AdGFP group, Unpaired *t*-test (B), One-way ANOVA followed by Bonferroni’s post hoc (C, D).

**Fig. 8:**
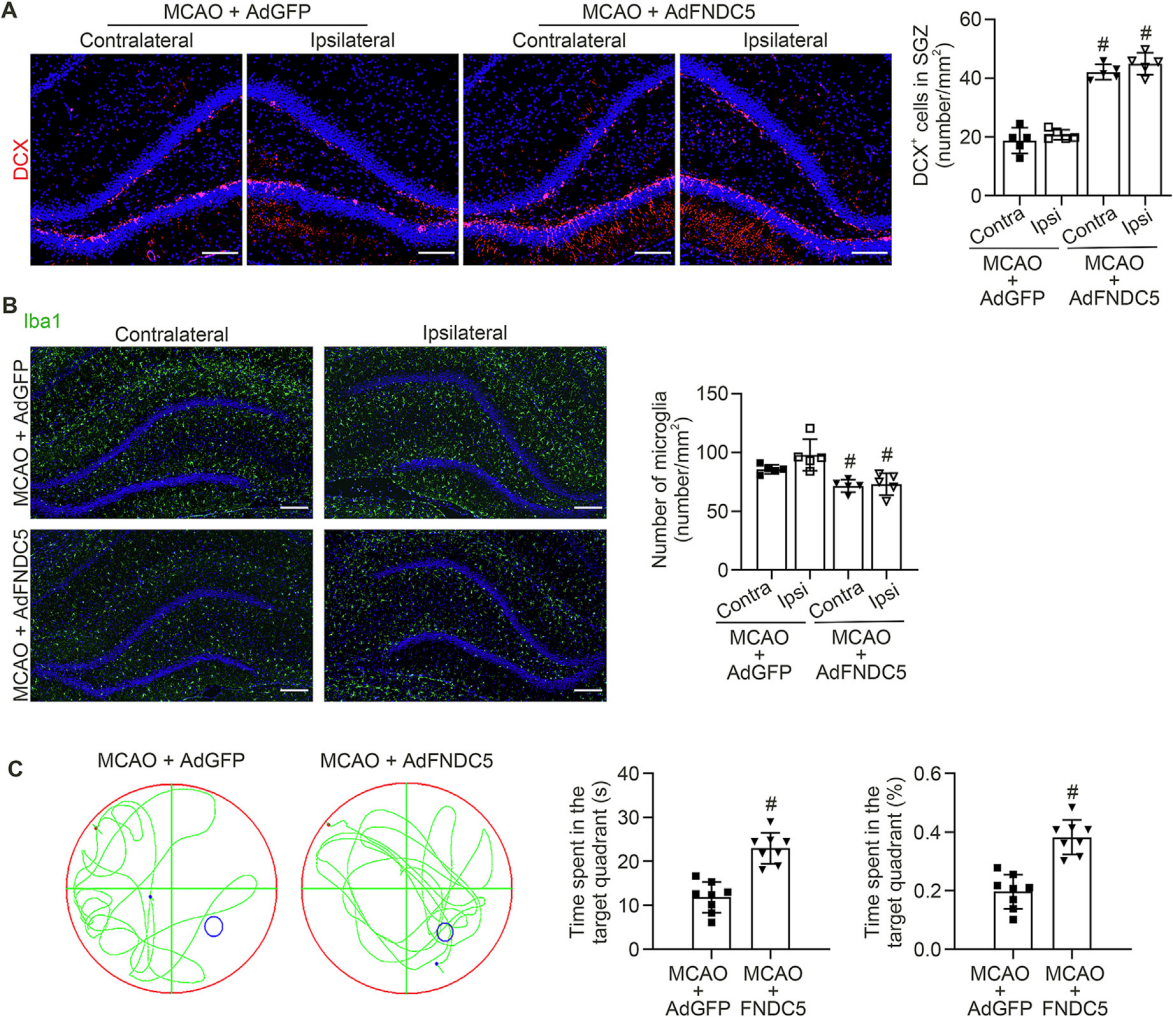
Peripheral FNDC5/irisin overexpression promoting neurogenesis and alleviating neuroinflammation in hippocampus after stroke in rats. (A) Representative images of DCX immunostaining in the bilateral hippocampi SGZ of MCAO + AdGFP and MCAO + AdFNDC5 groups 28 days post-operation, and quantitative analysis of DCX^+^ cell number in the bilateral hippocampi SGZ (*n* = 5 per group). Scale bar, 200 μm. Three different sections and three fields in each section are used for DCX^+^ cell number quantification per animal. (B) Representative images of Iba1 immunostaining in bilateral hippocampi of MCAO + AdGFP and MCAO + AdFNDC5 groups 28 days post-operation, and quantitative analysis of microglial count in the bilateral hippocampi (*n* = 5 per group). Scale bar, 250 μm. (C) Representative probe trial swimming traces and quantitative analysis of time spent in the target quadrant 28 days post-operation in MCAO + AdGFP and MCAO + AdFNDC5 groups (*n* = 8 per group). Contra, contralateral to the infarct; Ipsi, ipsilateral to the infarct; SGZ, subgranular zone. All data are presented as mean ± SD. #*p* < 0.05 versus MCAO + AdGFP group, One-way ANOVA followed by Bonferroni’s post hoc (A, B), Unpaired *t*-test (C).

To further investigate the role of the muscle–brain axis and the therapeutic potential of peripheral irisin in PSCI, rats were administered FNDC5-expressing adenoviral particles (AdFNDC5) or negative control GFP-expressing adenoviral particles (AdGFP) via caudal vein injection on days 8, 15, and 22 post-MCAO **(**Fig. 7**A)**. The MCAO + AdFNDC5 group exhibited significantly higher FNDC5 expression levels in the livers compared to the MCAO + AdGFP group (*p* < 0.05, Fig. 7**B**). Moreover, the MCAO + AdFNDC5 group demonstrated markedly elevated serum irisin levels (21.0 ng/mL ± 3.8 ng/mL) in contrast to the MCAO (5.6 ng/mL ± 2.8 ng/mL) and sham groups (12.8 ng/mL ± 2.0 ng/mL; all *p* < 0.05, Fig. 7**C**). Peripheral irisin overexpression significantly upregulated BDNF and DCX protein expression in the bilateral hippocampi compared to the MCAO group (*p* < 0.05, Fig. 7**D**). Additionally, the number of DCX^+^ neurons significantly increased in the SGZ of the bilateral hippocampi in the MCAO + AdFNDC5 group (ipsilateral, 42/mm^2^ ± 3/mm^2^; contralateral, 45/mm^2^ ± 4/mm^2^) compared to the MCAO + AdGFP group (ipsilateral, 21/mm^2^ ± 2/mm^2^; contralateral, 19/mm^2^ ± 4/mm^2^; all *p* < 0.05, Fig. 8**A**). The number of microglia was significantly fewer in the bilateral hippocampi in the MCAO + AdFNDC5 group (ipsilateral, 73/mm^2^ ± 9/mm^2^; contralateral, 71/mm^2^ ± 5/mm^2^) than the MCAO + AdGFP group (ipsilateral, 98/mm^2^ ± 14/mm^2^; contralateral, 86/mm^2^ ± 4/mm^2^; *p* < 0.05, Fig. 8**B**). In the Morris water maze test, the MCAO + AdFNDC5 group exhibited substantial cognitive enhancement during the probe trial, spending more time in the target quadrant (22.9 s ± 3.5 s) compared to the MCAO group (12.7 s ± 3.7 s) or the MCAO + AdGFP group (11.8 s ± 3.5 s; all *p* < 0.05, Fig. 8**C**).

In addition, we compared the effects of exercise and FNDC5 overexpression on post-stroke cognition in rats. There was no significant difference between these two groups regarding serum irisin levels (24.7 ng/mL ± 4.4 ng/mL vs. 21.0 ng/mL ± 3.8 ng/mL), DCX^+^ neuron counts (47/mm^2^ ± 2/mm^2^ vs. 45/mm^2^ ± 4/mm^2^), and time spent in the targeted quadrant in Morris water maze (23.0 s ± 3.8 s vs. 22.9 s ± 3.5 s; all *p* > 0.05, **Fig. S8**).

## Discussion

In this study, we demonstrated that patients with chronic stroke exhibited significantly reduced CSA and increased fat fraction in the hemiplegic thigh, along with a corresponding decrease in serum irisin levels. Serum irisin levels in stroke patients were positively correlated with both muscle CSA and cognitive performance. Subsequently, we replicated and expanded upon these results in cynomolgus monkey and rat stroke models. We observed that muscle atrophy in the paretic limb was associated with a downregulation of the FNDC5/irisin/BDNF axis, a decrease in immature neurons in the bilateral hippocampi, and cognitive impairment. Importantly, targeted restoration of the FNDC5/irisin/BDNF axis through physical exercise or peripheral irisin overexpression led to a significant increase in BDNF expression and immature neurons in the bilateral hippocampi, ultimately improving PSCI in rats. Overall, our findings suggest a mechanistic connection in which stroke-induced muscle loss disrupts the FNDC5/irisin/BDNF pathway, eventually worsening PSCI. This highlights the potential therapeutic benefit of enhancing this axis through exercise or overexpression.

Clinically, muscle thickness is significantly reduced on the paretic side during the first 3 months after a stroke [27]. Lean muscle mass and volume are also lower on the paretic side for more than 6 months after a stroke [28]. Similarly, mice and rats experience weight loss and muscle atrophy as early as 7 days after a stroke [29,30]. Various pathogenic mechanisms contribute to hemiplegic muscle atrophy after a stroke, including immobilization, impaired feeding, sympathetic activation, inflammation, and denervation [31]. In our study, mDIXON-QUANT MRI confirmed paretic muscle atrophy and fat infiltration in patients 3–6 months post-stroke.

Accumulating evidence indicates that muscle functions as an endocrine organ by releasing myokines. FNDC5/irisin has been identified as a novel myokine that facilitates communication between skeletal muscle and the brain, exerting positive effects on brain health and cognition [6,32]. Muscle mass and exercise correlate positively with FNDC5/irisin expression levels through the upregulation of PGC1α/FNDC5 in skeletal muscle [13,15]. Cross-sectional studies have indicated a positive association between serum irisin levels and cognition in patients with mild cognitive impairment, and these levels are significantly reduced in patients with vascular dementia [33,34]. However, changes of serum irisin levels post-stroke and their connection to PSCI remain unclear. In our investigation, we established that serum irisin levels were significantly lower in chronic stroke patients compared to healthy controls and were positively linked to both the CSA of the paretic thigh and cognitive function for the first time. In addition to post-stroke muscle atrophy, exercise intensity and nutritional status may affect circulating irisin levels. In this study, patients with cachexia-inducing conditions such as heart failure, liver failure, renal failure, or malignancy were excluded, and all patients had normal swallowing function at discharge, which minimized the potential risk of malnutrition and nutritional bias. Indeed, albumin levels of all patients were >35 g/L during hospitalization and follow-up, suggesting that chronic malnutrition was unlikely in the included patients. Moreover, participants were all instructed to refrain from physical exercise on the day prior to fasting blood collection. Previous studies have shown that circulating irisin peaks approximately 6 h after exercise and returns to baseline within 24 h [35]. Therefore, although residual confounding by lifestyle factors cannot be entirely excluded, the influence of physical activity and nutritional status on the present results was likely minimal. Several factors may account for robust association between serum irisin levels and post-stroke cognition in this study. First, the biological association between irisin and cognitive performance after stroke may be intrinsically strong, which could contribute to a higher explanatory power. Second, the relatively small and homogeneous sample may have reduced variability, thereby inflating the correlation coefficient. After adjustment for infarct volume, serum irisin levels remained positively associated with post-stroke cognition. Nevertheless, we acknowledge that the strength of this correlation may be overestimated in the present dataset and future studies with larger sample sizes and multivariable adjustment are required to validate this relationship. Taking together, our preliminary clinical study suggest that hemiplegic muscle loss leads to reduced irisin levels, potentially contributing to PSCI.

Irisin exerts its effects on the brain via promoting BDNF expression in the hippocampus [12]. To investigate the impact of reduced muscle-derived irisin levels post-stroke on hippocampal BDNF expression, we developed stroke models in animals. Cynomolgus monkeys, which share genetic and structural similarities with humans, were used to simulate clinical conditions by examining skeletal muscle atrophy and FNDC5/irisin/BDNF axis expression post-chronic stroke. Twelve weeks after stroke, cynomolgus monkeys had left-over hemiparesis and reduced extremity movement and showed muscle atrophy on the paretic side. PGC-1α/FNDC5 protein levels were significantly decreased in atrophic muscle, along with lower serum and CSF irisin levels. This decline in FNDC5/irisin was accompanied by a significant reduction in BDNF expression levels in the bilateral hippocampi. Notably, in our monkey stroke model, the hippocampus was distant from the primary infarct, indicating that the decreased BDNF levels in the hippocampus was little possibly attributed to ischemia or *peri*-infarct injury. Moreover, our research revealed a decline in immature neurons in the bilateral hippocampi of stroke-afflicted monkeys. BDNF is a well-established promoter of hippocampal neurogenesis [26]. Peripheral irisin crosses the BBB and promotes BDNF synthesis in the bilateral hippocampus. Downregulation of FNDC5/irisin/BDNF axis could account for the synchronous decline in DCX^+^ neuronal generation. Although other studies have reported enhanced neurogenesis after stroke, MCAO typically causes an infarct in the striatum and basal ganglia and often induces neurogenesis in the subventricular zone (SVZ) close to the infarct rather than in the hippocampal SGZ [36]. Rudolph et al. observed an increase in BrdU-positive neurons in the hippocampal SGZ six days post-MCAO in mice, but followed by a subsequent decrease [37]. In our study, spanning a longer observation period, we observed a reduction in immature neurons in the hippocampal SGZ 12 weeks post-stroke in cynomolgus monkeys. Neurogenesis in hippocampus modulates cognition by modifying the dentate gyrus network [38]. Therefore, reduced BDNF expression and neurogenesis in the bilateral hippocampi may aggravate PSCI. These findings suggest that chronic stroke-induced hemiplegic muscle atrophy contributes to the downregulation of the FNDC5/irisin/BDNF axis and diminished neurogenesis in the hippocampus, potentially worsening PSCI.

Physiologically, irisin is released from muscle fibers into the circulation in response to exercise [14,15]. Exercise protects mice with Alzheimer’s disease against cognitive impairment mediated by FNDC5/irisin [10]. Peripherally delivered FNDC5/irisin can cross the blood–brain barrier [10,11]. However, the therapeutic potential of the FNDC5/irisin/BDNF axis in PSCI remains uncertain. Our study, similar to observations in cynomolgus monkeys, revealed that rats displayed muscle atrophy and disruption of the FNDC5/irisin/BDNF axis 28 days after a stroke. To further investigate the role of the FNDC5/irisin/BDNF axis in PSCI and assess its therapeutic potential against PSCI, rats underwent physical exercise training 7 days after the stroke. Our results showed that physical exercise ameliorated muscle atrophy, increased PGC-1α/FNDC5 expression levels in skeletal muscle of both affected and unaffected limbs, and significantly increased serum irisin levels. The release of irisin due to exercise promoted BDNF expression and neurogenesis in the hippocampus, ultimately enhancing cognition in rats with stroke. These findings are consistent with those of clinical trials demonstrating the positive impact of physical exercise training on cognition in chronic patients [39]. In stroke rats, anti-FNDC5 blocked the protective effects of exercise on cognition, indicating that intact FNDC5/irisin signaling is required for exercise-induced cognitive improvement after stroke.

Another group of stroke rats received intravenous administration of an FNDC5-expressing adenoviral vector to induce peripheral FNDC5/irisin overexpression. Prior work has demonstrated that hepatic FNDC5 overexpression via tail vein injection of an FNDC5-overexpressing adenoviral vector leads to elevated serum irisin protein levels and improves cognition in Alzheimer’s disease mouse models [11,12]. Previous studies have shown that irisin treatment in the acute phase reduces infarct volume and improves neurological function in rodents [40]. To exclude the potential effects of peripheral irisin delivery on the primary infarct, we administered an AdFNDC5 injection on the seventh day after stroke, when the primary infarct was stable and skeletal muscle atrophy had occurred [29,30]. Our results confirmed that peripheral FNDC5/irisin overexpression significantly increased BDNF expression levels and the number of immature neurons in the hippocampus, ultimately ameliorating PSCI 28 days after stroke in rats. These findings further support that the downregulation of the FNDC5/irisin/BDNF axis aggravates PSCI. Both exercise and peripheral FNDC5/irisin overexpression upregulate this axis, providing neuroprotective effects against PSCI in rats. Enhancing the FNDC5/irisin pathway via exercise or by viral overexpression yields comparable elevations in circulating irisin, hippocampal neurogenesis, and spatial memory performance under our experimental conditions. Nevertheless, in clinical practice, exercise interventions are more feasible than gene-mediated FNDC5 overexpression.

Moreover, microglial activation was observed in hippocampus after stroke, indicating an enhanced inflammatory response. Importantly, both exercise and FNDC5/irisin overexpression attenuated this hippocampal inflammation, as evidenced by a significant reduction in microglial counts. These findings suggest that muscle-derived FNDC5/irisin may improve cognition not only by upregulating BDNF and enhancing neurogenesis but also by suppressing hippocampal inflammation. This is in line with previous studies reporting that irisin exerts anti-inflammatory effects in central nervous system diseases [41].

Post-stroke cognitive impairment is caused by the interplay of multiple mechanisms. Clinical studies have shown that lesion burden and strategic infarct locations associate with the risk of PSCI [2,42]. Recurrent stroke and cerebral small vessel disease further increase the risk of delayed PSCI [43,44]. Increasing evidence also highlights neuroinflammation as a critical driver of PSCI, with microglia playing a central role [45]. Beyond the *peri*-infarct region, both our previous work and studies by others have reported neuronal loss and microglial activation in remote areas such as the thalamus and hippocampus, a process termed secondary neurodegeneration. Alleviating secondary neurodegeneration significantly improves post-stroke cognition in both non-human primates and rodents [3,46,47]. This study extends current knowledge of the mechanisms of PSCI, identifying muscle atrophy–induced disruption of the muscle–brain axis as a contributing factor.

Our integrative, multi-species approach highlights that stroke-induced muscle atrophy results in the downregulation of the FNDC5/irisin/BDNF axis, ultimately exacerbating PSCI. However, this study had certain limitations. The sample size in cynomolgus monkey experiments was limited due to ethical considerations and substantial cost. We exclusively utilized male animals in our investigation to mitigate sex-related variability in stroke prognosis; future studies should encompass female subjects for broader generalizability. Additionally, we did not examine whether exercise upregulated the FNDC5/irisin/BDNF axis in stroke cynomolgus monkeys as observed in rats. Another limitation is that FNDC5 overexpression and inhibition in rats were achieved through hepatic overexpression (AdFNDC5) and systemic administration of a neutralizing antibody, rather than muscle-specific targeting, which may not fully reflect the physiological origin of circulating irisin. Our interventions (exercise / anti-FNDC5 administration / AdFNDC5 administration) were initiated on postoperative day 7 and cognition was evaluated at a single time-point (postoperative day 28) in rats, which precluded detailed characterization of temporal dynamics. Future studies will employ repeated longitudinal assessments (acute–subacute–chronic) with mixed-effects modeling to determine onset, trajectory, and durability of treatment effects. The sample size of our clinical study was relatively limited. Despite this limitation, the significant association between serum irisin levels and post-stroke cognition was still observed. The cross-sectional nature of our clinical study precludes establishing temporal order or causal direction. Further research involving larger prospective cohorts of stroke patients is necessary to investigate temporal changes in peripheral irisin levels, its relation with post-stroke cognition, and to evaluate the impact of exercise on PSCI.

## Conclusions

Overall, our study demonstrates that stroke-induced hemiplegic muscle atrophy leads to the downregulation of the FNDC5/irisin/BDNF axis, contributing to impaired hippocampal neurogenesis, activated neuroinflammation and worsening of PSCI. Restoration of this muscle–brain axis through exercise or peripheral irisin overexpression effectively improves cognitive function, providing a new approach for post-stroke rehabilitation.

## Compliance with ethics requirements

All Institutional and National Guidelines for the care and use of animals were followed. Cynomolgus monkey study was reviewed and approved by the institutional Animal Care and Use Committee of Guangdong Landau Biotechnology Co., Ltd. (LDACU20190215-01). SD rat study was reviewed and approved by the institutional Animal Care and Use Committee of Sun Yat-Sen University (SYSU-IACUC-2023–001422).

## Compliance with ethics requirements

All procedures followed were in accordance with the ethical standards of the responsible committee on human experimentation (institutional and national) and with the Helsinki Declaration of 1975, as revised in 2008. It was approved by the Independent Ethics Committee of the First Affiliated Hospital of Sun Yat-sen University (FAH-SYSU-IEC-[2023]838). Informed consent was obtained from all patients for being included in the study.

## Funding

This research was funded by the 10.13039/501100001809Natural Science Foundation of China (Key program, 82130035; General program: 82371308; Youth program: 82201436), the Natural Science Foundation of Guangdong Province (2024A1515012877), the Guangzhou Science-Brain Project (2024A04J4627), Guangdong Provincial Clinical Research Center for Neurological Diseases (2020B1111170002), Guangdong Province International Cooperation Base for Early Intervention and Functional Rehabilitation of Neurological Diseases (2020A0505020004), Guangzhou Major Difficult and Rare Diseases Project (2024MDRD02), Guangdong Provincial Engineering Center for Major Neurological Disease Treatment, Guangdong Provincial Translational Medicine Innovation Platform for Diagnosis and Treatment of Major Neurological Disease, Guangzhou Clinical Research and Translational Center for Major Neurological Diseases.

## Declaration of competing interest

The authors declare that they have no known competing financial interests or personal relationships that could have appeared to influence the work reported in this paper.
