# Supplementary material for: Targeting the muscle-brain axis to improve post-stroke cognition via the FNDC5/irisin/BDNF pathway
Source: J Adv Res. 2025 Oct 25;85:853–68. doi: 10.1016/j.jare.2025.10.027 (PMC13316544; doi:10.1016/j.jare.2025.10.027)
Supplement: Supplementary Data 1 [file mmc1.pdf]

Targeting the muscle-brain axis to improve post-stroke cognition via the FNDC5/irisin/BDNF pathway

Jiating Wei[a,b,c,1], Yuangui Cai[a,b,c,1], Zimu Jiang[a,b,c,1], Jia Xie[d], Dingxiang Xie[d], Fubing Ouyang[a,b,c], Jianle Li[a,b,c], Zhiyi Xiong[a,b,c], Xiya Long[a,b,c], Miaoxian Yang[a,b,c], Lisi Zha[a,b,c], Yingxin He[a,b,c], Weixian Huang[a,b,c], Jinsheng Zeng[a,b,c*]

a Department of Neurology, The First Affiliated Hospital of Sun Yat-sen University, Guangzhou, Guangdong, 510080, People's Republic of China.

b Guangdong Provincial Key Laboratory of Diagnosis and Treatment of Major Neurological Diseases, Guangzhou, Guangdong, 510080, People's Republic of China.

c National Key Clinical Department and Key Discipline of Neurology, Guangzhou, Guangdong, 510080, People's Republic of China.

d Department of Radiology, The First Affiliated Hospital of Sun Yat-Sen University, Guangzhou, Guangdong, 510080, People's Republic of China.

[1] These authors share the first co-authorship.

***Corresponding author:** Jinsheng Zeng

Address: Department of Neurology, The First Affiliated Hospital of Sun Yat-sen University, No.58 Zhongshan Er Road, Guangzhou, Guangdong, 510080, People's Republic of China

Phone: +86-20-87755766-8253

E-mail: zengjsh@mail.sysu.edu.cn

Running title : muscle-brain axis AND PSCI

Supplementary table

Table S1 Clinical characteristics of stroke patients and health control

Characteristics	Health control (n = 24)	Stroke patients (n = 22)	P
Demography			
Age, years; mean ± SD	64.8 ± 8.7	65.1 ± 10.4	0.93
Male, sex; n (%)	13 (54.2)	18 (81.8)	0.06
Education, years; mean ± SD	10.6 ± 3.8	9.4 ± 3.5	0.26
Vascular risk factors			
Hypertension; n (%)	12 (50.0)	14 (63.6)	0.39
Diabetes; n (%)	6 (25.0)	9 (40.9)	0.35
Dyslipidemia; n (%)	18 (75.0)	11 (50.0)	0.13
Smoke; n (%)	8 (33.3)	9 (40.9)	0.76
Alcohol consumption; n (%)	5 (20.8)	4 (18.2)	0.99
Dysphagia; n (%)	0 (0)	0 (0)	>0.99
Baseline albumin, g/L; mean ± SD	43.9±1.2	41.2±4.2	0.08
Follow-up albumin, g/L; mean ± SD	–	42.2±2.6	
Cognition test			
MoCA, score; mean ± SD	25.6 ± 2.0	20.6 ± 5.8	**<0.01**
TMT-A, s; mean ± SD	67.1±21.0	109.4±85.3	**<0.01**
TMT-B, s; mean ± SD	270.0 ± 82.0	414.3 ± 197.5	**<0.01**
AVLT-H, number; mean ± SD	7.5 ± 2.0	4.9 ± 2.9	**<0.01**

MoCA, Montreal Cognitive Assessment; TMT-A, Trail Making Test A; TMT-B, Trail Making Test B; AVLT-H, Auditory Verbal Learning Test-Huashan Version.

Table S2 Association between serum irisin level and cognitive function with or without adjustment for infarct volume

**Model**	B (95% CI)	beta	*P*
**MoCA**			
Simple linear regression	0.092 [0.059, 0.125]	0.793	<0.001
Adjusted for infarct volume	0.089 [0.056, 0.123]	0.770	<0.001
**TMT-A**			
Simple linear regression	-0.915 [-1.583, -0.247]	-0.538	0.010
Adjusted for infarct volume	-0.813 [-1.435, -0.191]	-0.479	0.013
**TMT-B**			
Simple linear regression	-2.540 [-4.525, -0.555]	-0.512	0.015
Adjusted for infarct volume	-2.435 [-4.478, -0.391]	-0.491	0.022
**AVLT-H**			
Simple linear regression	0.029 [0.006, 0.052]	0.500	0.018
Adjusted for infarct volume	0.028 [0.004, 0.052]	0.481	0.026

MoCA, Montreal Cognitive Assessment; TMT-A, Trail Making Test A; TMT-B, Trail Making Test B; AVLT-H, Auditory Verbal Learning Test-Huashan Version.

Supplementary Figures

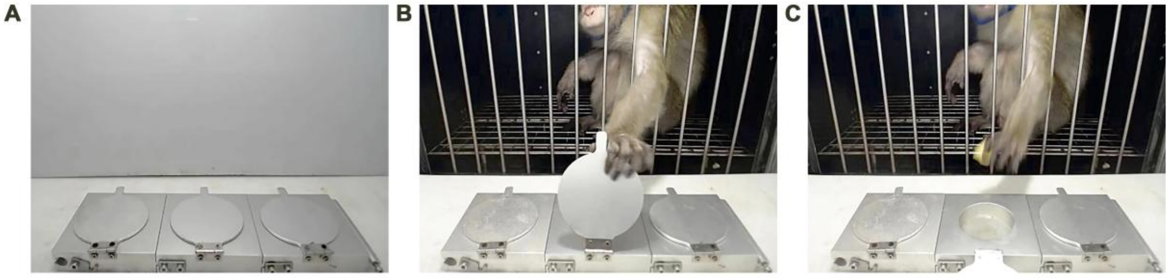

Fig. S1. Schematic of delayed response test in cynomolgus monkeys. Cynomolgus monkeys were shown which well the bait was put in. Then, the opaque Plexiglas cover slid down **(A)**. After delay, cover slid up and cynomolgus monkeys were required to retrieve the hidden food from one of the three wells **(B, C)**.

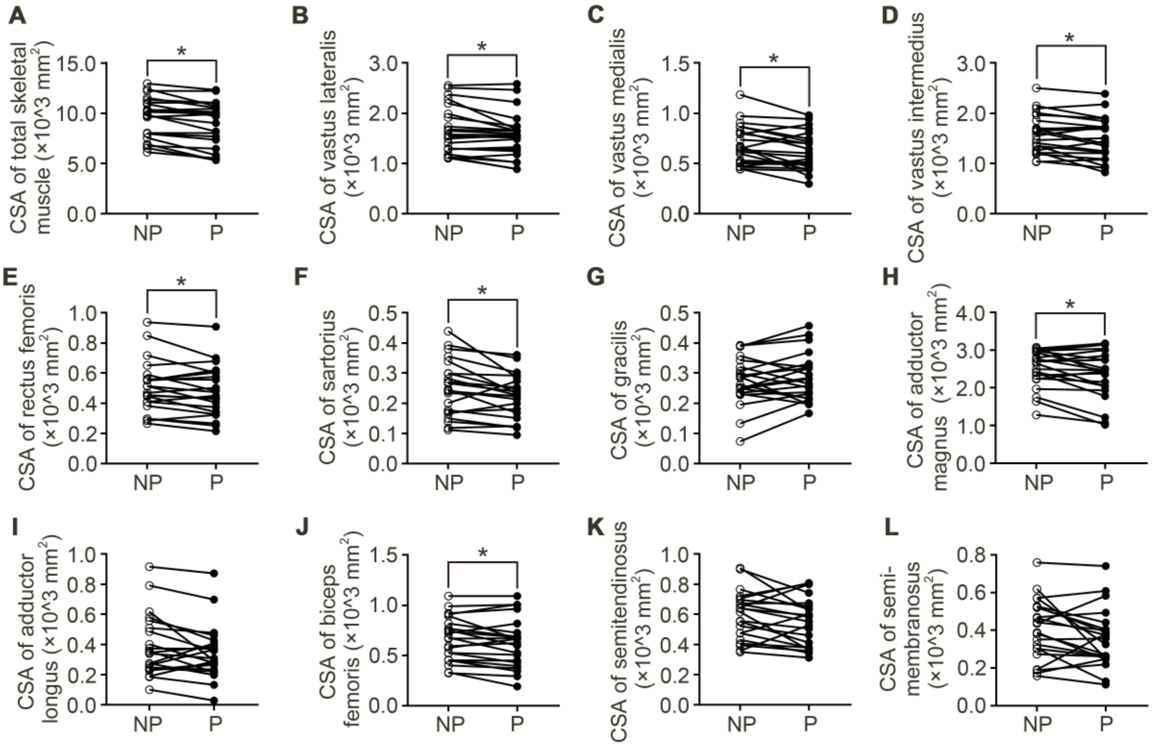

Fig. S2. Hemiplegic muscle atrophy in patients 3 months to 6 months after stroke. Analysis of CSA of total skeletal muscle **(A)**, vastus lateralis **(B)**, vastus medialis **(C)**, vastus intermedius **(D)**, rectus femoris **(E)**, sartorius **(F)**, gracilis **(G)**, adductor magnus **(H)**, adductor longus **(I)**, biceps femoris **(J)**, semitendinosus **(K)**, and semimembranosus **(L)** between paretic and nonparetic thigh in patients 3 to 6 months after stroke (n = 22). CSA, cross-sectional area; NP, nonparetic; P, paretic. *P < 0.05 versus nonparetic thigh, Paired t-test.

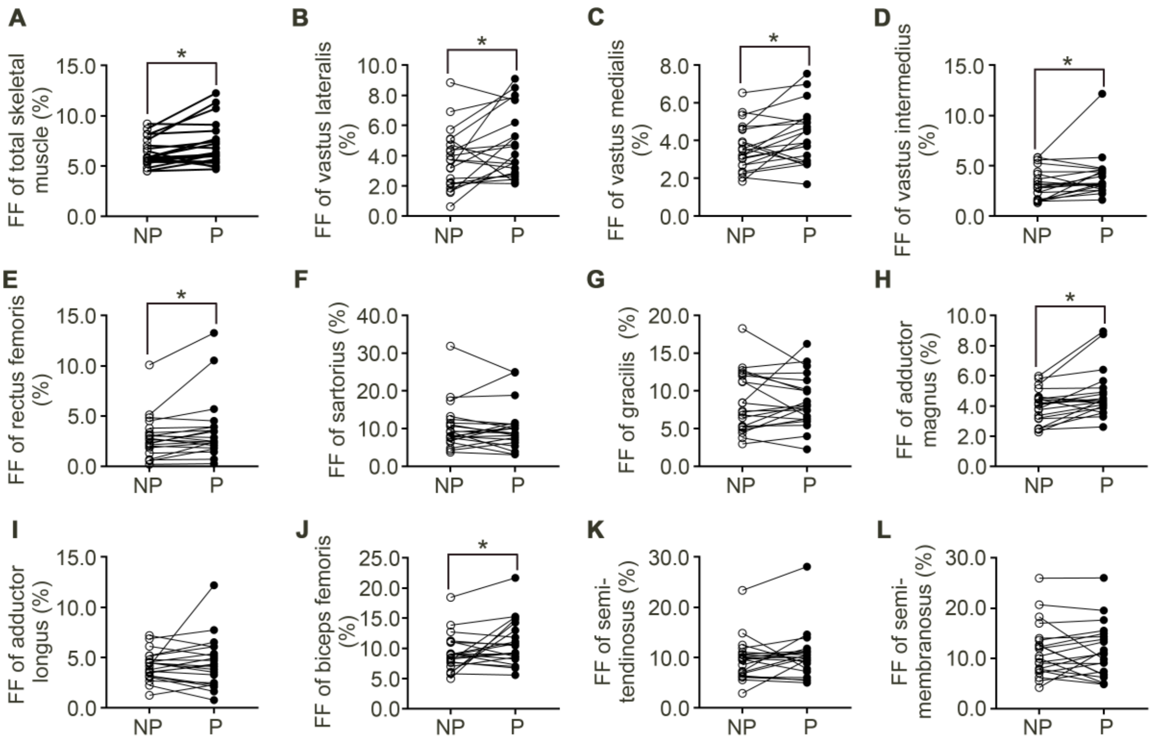

Fig. S3. Increased fat fraction of hemiplegic muscle in patients 3 to 6 months after stroke. Analysis of CSA of total skeletal muscle **(A)**, vastus lateralis **(B)**, vastus medialis **(C)**, vastus intermedius **(D)**, rectus femoris **(E)**, sartorius **(F)**, gracilis **(G)**, adductor magnus **(H)**, adductor longus **(I)**, biceps femoris **(J)**, semitendinosus **(K)**, and semimembranosus **(L)** between paretic and nonparetic thigh in patients 3 to 6 months after stroke (n = 22). FF, fat fraction; NP, nonparetic; P, paretic. *$P < 0.05$ versus nonparetic thigh, Paired t-test.

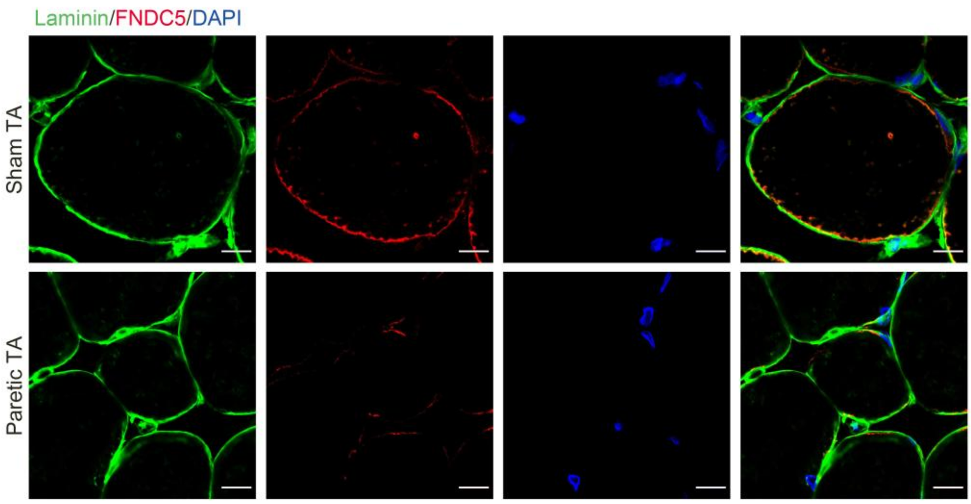

Fig. S4. Decreased FNDC5 protein expression in TA muscle 12 weeks after MCAO in cynomolgus monkeys. Representative images of laminin (green), FNDC5 (red) and DAPI (blue) co-immunostaining of TA muscle 28 days after MCAO or sham operation in cynomolgus. Scale bar, 10 μm. TA, tibialis anterior.

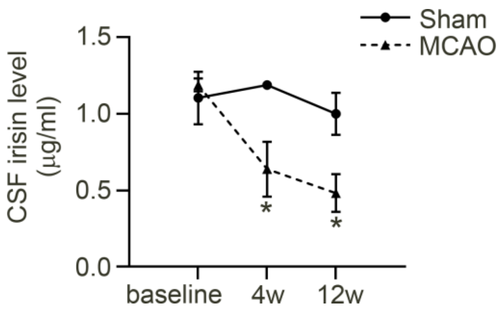

Fig. S5. Reduced CSF irisin levels 12 weeks after stroke in cynomolgus monkeys. Temporal change of CSF irisin level in the MCAO or sham group ($n = 3$ per group). $*p < 0.05$ versus baseline, Two-way ANOVA, followed by Bonferroni's post hoc.

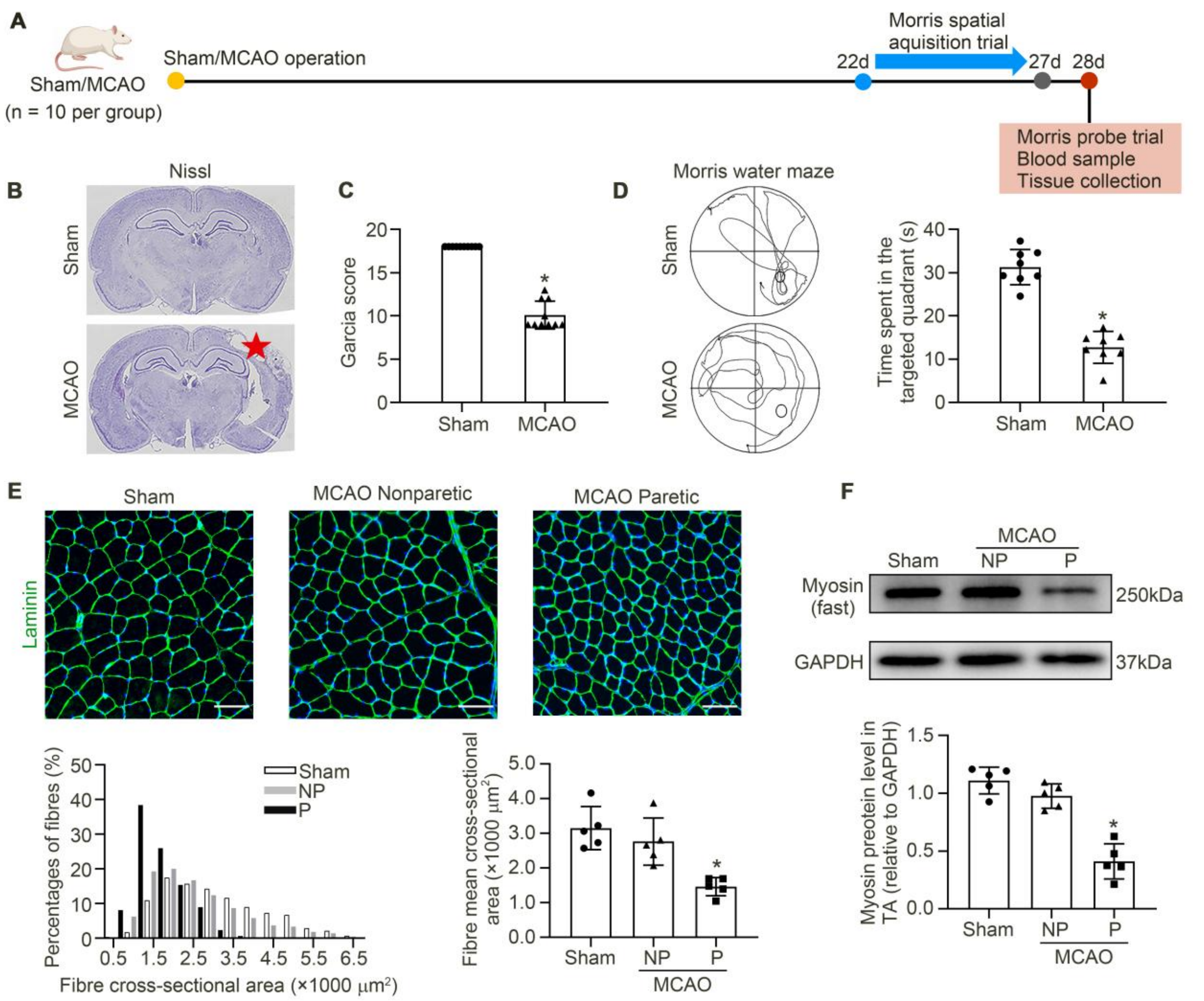

Fig. S6. Cognitive impairment and paretic muscle atrophy 28 days after stroke in rats. **(A)** Outline of the rat experimental design. **(B)** Representative images of Nissl staining. The star indicates infarct. **(C)** Neurological deficits evaluated by Garcia scale 24h after MCAO or sham operation in rats (*n* = 10 per group). **(D)** Representative swimming traces of probe trial and quantitative analysis of time spent in the targeted quadrant 28 days after MCAO or sham operation (*n* = 8 per group). **(E)** Representative images of laminin immunofluorescence staining of TA muscle 28 days after MCAO or sham operation, and fibre size distribution and fibre mean CSA of TA muscle calculated by laminin immunostaining. More than 200 fibres are randomly used for CSA quantification per animal (*n* = 5 per group). Scale bar, 100 μm. **(F)** Representative Westernblot band and quantitative analysis of fast myosin protein expression in TA muscle 28 days after MCAO or sham operation (*n* = 5 per group). CSA,

cross-sectional area; NP, nonparetic; P, paretic; TA, tibialis anterior. All data are presented as mean ± SD. *$p < 0.05$ versus sham, One-way ANOVA followed by Bonferroni's post hoc (E, F), Unpaired t-test (C, D).

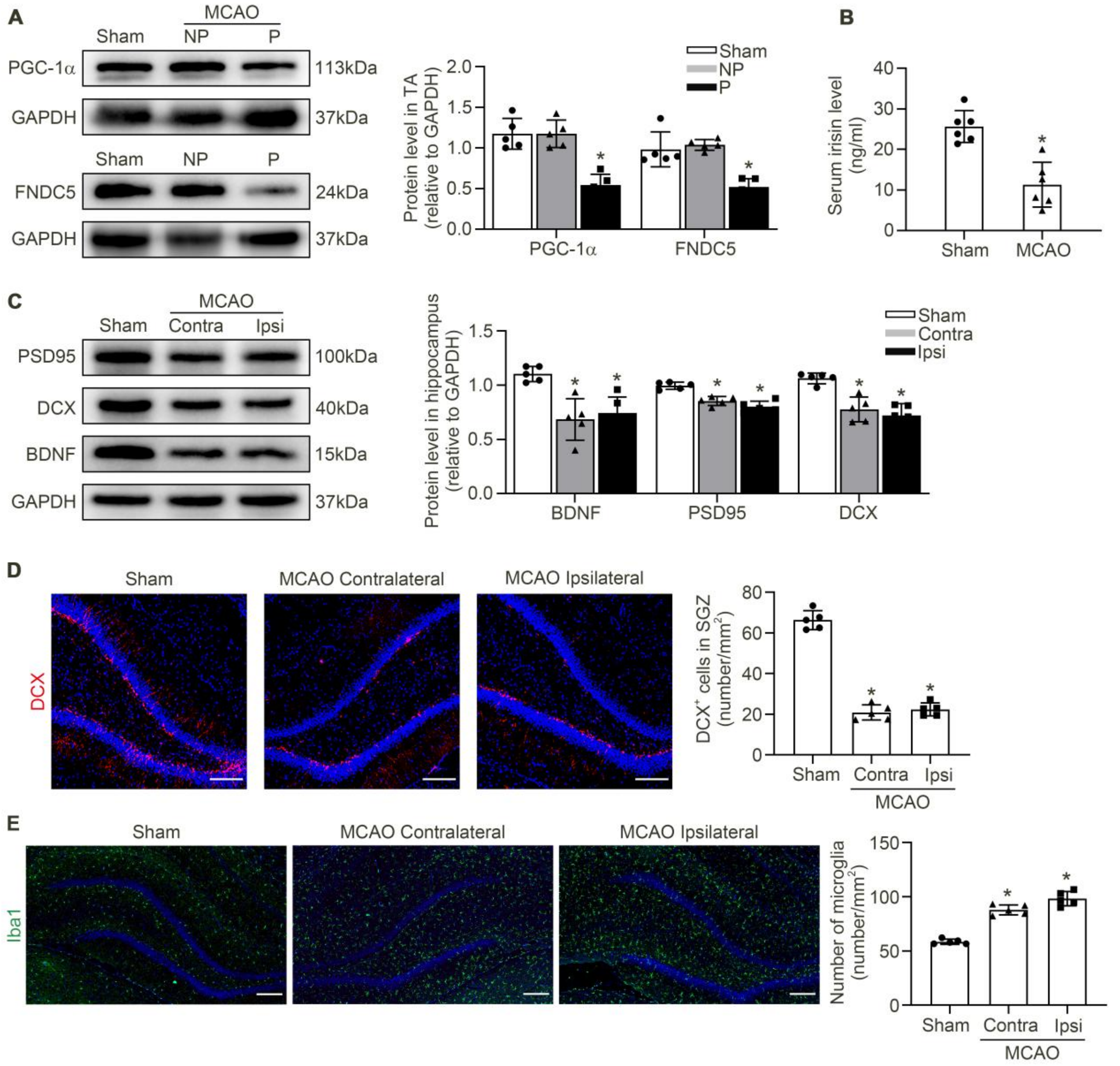

Fig. S7. PGC-1α/FNDC5/Irisin/BDNF downregulation 28 days after stroke in rats. **(A)** Representative Westernblot band and quantitative analysis of PGC-1α/FNDC5 protein expression levels in TA muscle 28 days after MCAO or sham operation (n = 5 per group). **(B)** Quantitative analysis of serum irisin level 28 days after MCAO or sham operation (n = 6 per group). **(C)** Representative Westernblot band and quantitative analysis of BDNF, PSD95 and DCX protein expression level in bilateral hippocampus 28 days after MCAO or sham operation (n = 5 per group). **(D)** Representative images of DCX immunostaining in bilateral hippocampus SGZ 28 days after MCAO or sham operation, and quantitative analysis of DCX^+ cell number in bilateral hippocampus SGZ (n = 5 per group). Three different sections

and 3 fields in each section are used for DCX^+ cell number quantification per animal. Scale bar, 200 µm. **(E)** Representative images of Iba1 immunostaining in the bilateral hippocampi 28 days after MCAO or sham operation, and quantitative analysis of microglial count in the bilateral hippocampi (n = 5 per group). Scale bar, 250 µm. Contra, contralateral to the infarct; Ipsi, ipsilateral to the infarct; NP, nonparetic; P, paretic; SGZ, subgranular zone; TA, tibialis anterior. All data are presented as mean ± SD. $*p < 0.05$ versus sham, One-way ANOVA followed by Bonferroni's post hoc (A, C, D, E), Unpaired t-test (B).

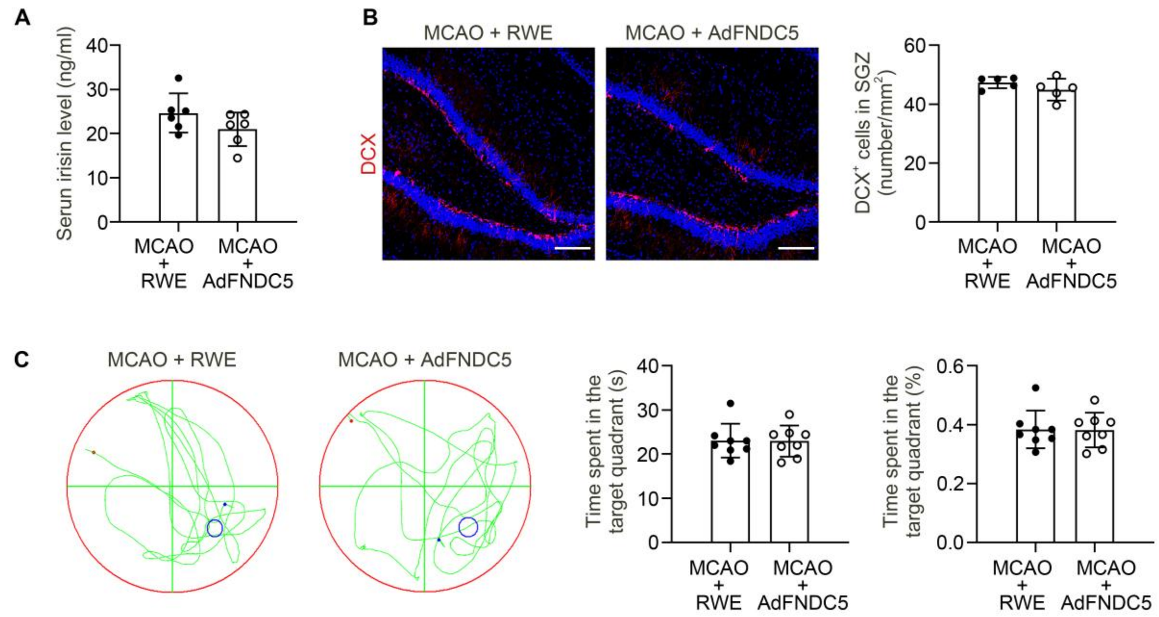

Fig. S8. No significant difference in the effects of exercise and FNDC5 overexpression on post-stroke cognition in rats. **(A)** Quantitative analysis of serum irisin level 28 days after operation in the MCAO + Exercise and MCAO + AdFNDC5 groups (n = 6 per group). **(B)** Representative images of DCX immunostaining in bilateral hippocampus SGZ 28 days after operation in the MCAO + Exercise and MCAO + AdFNDC5 groups, and quantitative analysis of DCX^+ cell number in bilateral hippocampus SGZ (n = 5 per group). Three different sections and 3 fields in each section are used for DCX^+ cell number quantification per animal. Scale bar, 200 μm. **(C)** Representative probe trial swimming traces and quantitative analysis of time spent in the target quadrant 28 days post-operation in MCAO + Exercise and MCAO + AdFNDC5 groups (n = 8 per group). RWE, running wheel exercise. All data are presented as mean ± SD. Unpaired t-test.
